# Geographic microtargeting of social assistance with high-resolution poverty maps

**DOI:** 10.1073/pnas.2120025119

**Published:** 2022-08-01

**Authors:** Isabella S. Smythe, Joshua E. Blumenstock

**Affiliations:** ^a^School of International and Public Affairs, Columbia University, New York, NY 10027;; ^b^School of Information, University of California, Berkeley, CA 94720

**Keywords:** poverty, targeting, satellite imagery, Nigeria

## Abstract

Hundreds of millions of poor families receive some form of targeted social assistance. Many of these antipoverty programs involve some degree of geographic targeting, where aid is prioritized to the poorest regions of the country. However, policy makers in many low-resource settings lack the disaggregated poverty data required to make effective geographic targeting decisions. Using several independent datasets from Nigeria, this paper shows that high-resolution poverty maps, constructed by applying machine learning algorithms to satellite imagery and other nontraditional geospatial data, can improve the targeting of government cash transfers to poor families. Specifically, we find that geographic targeting relying on machine learning–based poverty maps can reduce errors of exclusion and inclusion relative to geographic targeting based on recent nationally representative survey data. This result holds for antipoverty programs that target both the poor and the extreme poor and for initiatives of varying sizes. We also find no evidence that machine learning–based maps increase targeting disparities by demographic groups, such as gender or religion. Based in part on these findings, the Government of Nigeria used this approach to geographically target emergency cash transfers in response to the COVID-19 pandemic.

## Significance

Many antipoverty programs use geographic targeting to prioritize benefits to people living in specific locations. This paper shows that high-resolution poverty maps, constructed with machine learning algorithms from satellite imagery, can improve the geographic targeting of benefits to the poorest members of society. This approach was used by the Nigerian government to distribute benefits to millions of the extreme poor. As high-resolution poverty maps become globally available, these results can inform the design and implementation of social assistance programs worldwide.

Hundreds of millions of poor and vulnerable families benefit from some form of targeted social assistance (1). Just since the onset of the COVID-19 pandemic, over 3,300 new targeted social protection programs have been launched (1).

A key factor in the success of any antipoverty program is the degree to which it is accurately targeted (2). When truly poor families do not receive benefits (errors of exclusion) or when nonpoor families do receive benefits (errors of inclusion), this undermines the effectiveness of the policy (3).

Unfortunately, many governments in low- and middle-income countries (LMICs) lack recent, reliable data on where poverty is concentrated (4). While most LMICs have access to poverty data that provide comprehensive coverage at the largest administrative subdivision (e.g., the state level in Nigeria, comparable with the state level in the United States), coverage is far less complete at the third administrative subdivision (e.g., the ward level in Nigeria, comparable with municipalities in the United States). In Nigeria, the most recent Demographic and Health Survey (DHS) surveyed households in only 13.8% of wards. This problem is present across LMICs; in Peru, for example, 32.0% of the comparable administrative units were covered in the most recent DHS, and in Indonesia, just 16.1% were covered. In practice, this incomplete coverage means that a geographically targeted program must either rely on potentially inaccurate and outdated poverty maps or accept the efficiency losses of targeting larger administrative units.

In this paper, we ask the following question. Can fine-grained poverty maps, produced by applying deep learning algorithms to high-resolution satellite imagery, improve the accuracy of geographically targeted antipoverty programs? Our results are based on an analysis done in a high-stakes policy environment to help the Government of Nigeria determine its emergency COVID-19 response strategy.

Our main results evaluate different geographic targeting mechanisms available to the Nigerian government, which are similar to those used by policy makers in many LMICs. Specifically, we compare the targeting outcomes that would result from using high-resolution machine learning (ML)–based poverty maps with those that would result from using a recent nationally representative household survey (which we refer to as the survey-based “benchmark”). Both approaches are evaluated using a nationally representative survey of 22,110 Nigerian households that was independently collected and not used to train the ML-based approach or to determine the survey-based benchmark. By maintaining a clear separation between the data used to simulate targeting allocations and the data used to evaluate those allocations, we limit the scope for overfitting on the evaluation data.

We find that the ML-based poverty maps are at least as accurate as the benchmark in targeting benefits to the poor (i.e., those with consumption below the poverty line) and to the extreme poor (consumption below half the poverty line) in regions where benchmark data are available. We also document the main advantage of the ML-based maps, which is that they allow for accurate microtargeting in all administrative subdivisions of the country—including subdivisions where benchmark data do not exist. This is important because the survey benchmark does not contain data for 86.2% of Nigerian wards (the Admin-3 unit) and 18.5% of local government areas (LGAs; the Admin-2 unit). We document how the accuracy and complete coverage of the ML-based maps make it possible to design a more disaggregated geographic targeting policy than would be possible with survey data alone. This disaggregation directly translates to a higher fraction of benefits being allocated to the poor and the extreme poor.

In addition, we assess the fairness of ML-based targeting with respect to several different demographic subgroups. This is to address the concern that targeting approaches that are agnostic to recipients’ demographics may over- or undertarget certain groups, such as female-headed households (5, 6). Comparing the demographic parity of ML-based and survey-based targeting approaches along several dimensions, we find that ML-based targeting does not decrease fairness overall.

These results build on prior work that developed methods for the construction of high-resolution poverty maps (7–9). However, our focus is different and more practical. We take the output of prior work (the high-resolution poverty maps) as the input to our analysis and show how such maps can improve the outcomes of a real-world social assistance program. In January 2021, the Nigerian government chose this approach to guide the expansion of cash transfers to the urban poor (10); our hope is that this analysis can help encourage future efforts to integrate recent innovations in ML into humanitarian relief applications.

## Results

### Benefits of Disaggregation.

**Fig. 1.**
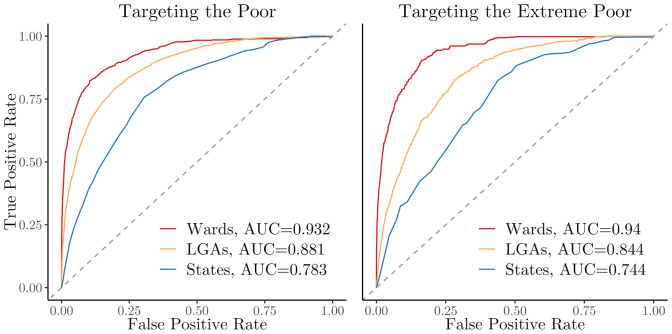
Targeting performance of policies at different administrative units. ROC curves show the performance of geographic targeting policies designed at the state (Admin-1), LGA (Admin-2), and ward (Admin-3) levels, where all households in a targeted administrative unit receive full benefits and households in untargeted units receive no benefits. The targeting of an administrative unit is determined based on the average wealth of the unit as calculated from NLSS data. True and false positive rates are calculated based on the portion of true poor households that are targeted, where true poverty status is determined based on the NLSS.

Our first intuitive result confirms prior work and highlights the value of geographic disaggregation in the design of geographic targeting policies. This analysis is shown in Fig. 1, where we compare targeting performance at different aggregation levels using hypothetical optimal targeting data. Optimal targeting is simulated by using the same survey data to both perform and evaluate targeting. This allows us to approximate how effectively targeting can be conducted when the true underlying distribution of poverty is known. However, it is important to note that this is a hypothetical exercise; no dataset exists that would allow for optimal targeting in practice.

Fig. 1, *Left* displays the receiver operating characteristic (ROC) curves where the objective is to provide benefits to the poor (daily consumption below $1.05); Fig. 1, *Right* provides the ROC curves for the objective of targeting the extreme poor (daily consumption below $0.57). Substantial increases in the area under the curve are produced as the targeting policy shifts from states (the largest administrative unit) to LGAs (the intermediate administrative unit) and from LGAs to wards (the smallest administrative unit). These findings are consistent with work done in other contexts to document the benefits of spatial disaggregation in geographic targeting (11–13); intuitively, programs targeting smaller administrative units are able to more precisely direct benefits to the poorest regions than programs targeting larger ones.

### Coverage and Accuracy of ML-Based Poverty Maps.

Our second set of results contrasts the coverage of ML-based poverty maps with survey-based alternatives and compares the accuracy of these two approaches at different spatial scales.

**Fig. 2.**
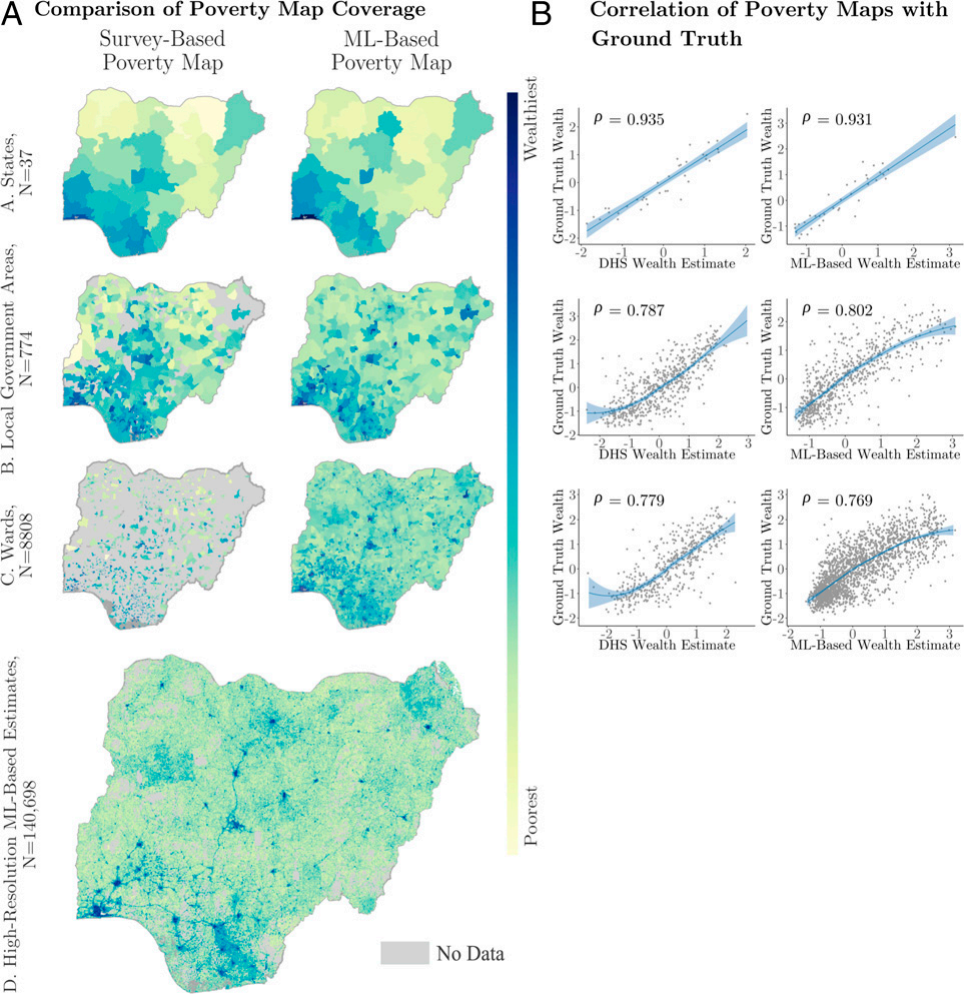
Coverage and correlations of ML and benchmark poverty maps to NLSS-estimated ground truth poverty maps. *A* compares the coverage and estimates of traditional survey-based poverty maps (*Left*) and ML-based poverty maps (*Right*) at the three different administrative levels: state (row A), LGA (row B), and ward (row C). Regions without data are shown in gray. Row D shows the high-resolution ML-based estimates prior to aggregation. For privacy reasons, high-resolution poverty estimates are not generated for grid cells with fewer than 10 inhabitants. *B* compares the ML and survey benchmark (the DHS) wealth estimates of each administrative unit against the NLSS ground truth estimate of that unit’s wealth. Pearson’s correlation coefficients are reported across all relevant units. Fewer observations exist in *B* because not all LGAs and wards contain households that were surveyed in the DHS. All correlations are significant at *P* = 0.001.

The difference in coverage between survey- and ML-based poverty maps is evident in Fig. 2*A*, which shows the two versions of Nigerian poverty maps side by side at different levels of geographic aggregation. Gray areas indicate administrative units where no surveys occurred in the benchmark dataset, a nationally representative DHS household survey of 40,427 households conducted in 2018. At the state level (row A), both maps have complete coverage; however, at the LGA level (row B), the survey-based map loses 18.5% of LGAs, and at the finest level (row C), surveys cover only 13.8% of all wards in Nigeria. A full tabulation of these results is also shown in the first two columns of Table 1.

**Table 1.** Coverage and accuracy of different approaches to constructing poverty maps in Nigeria

	No. of regions with estimates		Correlation with ground truth estimates	
	DHS	ML based	DHS	ML based
State-level correlations				
1) All states	37	37	0.935	0.931
			(0.877, 0.966)	(0.870, 0.964)
LGA-level correlations				
2) All LGAs	597	706	0.787	0.802
			(0.755, 0.816)	(0.774, 0.827)
3) > 30 ground truth households	333	371	0.839	0.863
			(0.804, 0.868)	(0.834, 0.887)
4) LGAs with DHS data		597		0.811
				(0.782, 0.837)
5) LGAs with no DHS data		109		0.713
				(0.606, 0.794)
6) > 30 ground truth households and no DHS		38		0.812
				(0.665, 0.898)
Ward-level correlations				
7) All wards	464	2,016	0.779	0.769
			(0.740, 0.812)	(0.751, 0.786)
8) > 20 ground truth households	95	242	0.894	0.870
			(0.844, 0.928)	(0.835, 0.897)
9) Wards with DHS data		464		0.792
				(0.756, 0.824)
10) Wards with no DHS data		1,552		0.759
				(0.737, 0.779)
11) > 20 ground truth households and no DHS		147		0.871
				(0.825, 0.905)

The first two columns indicate the number of administrative units for which data exist in the 2019 NLSS ground truth and the 2018 DHS (column 1) or the ML-based estimates (column 2). The last two columns indicate the Pearson correlation between wealth estimates generated from the ground truth (the NLSS) and the DHS (using representative imputation; column 3) or ML-based estimates (column 4). Correlations are measured across administrative units (i.e., not across households) using NLSS household weights for aggregation at the state level but not at the LGA or ward level. Different levels of spatial aggregation of wealth estimates are indicated. Rows 3, 6, 8, and 11 restrict analysis to administrative units where the NLSS ground truth contains a minimum of 20 to 30 households (to remove high-variance observations from the ground truth estimate). Rows 4 and 9 evaluate the ML-based estimates on the regions where DHS data exist; rows 5, 6, 10, and 11 evaluate the ML-based estimates on the subset of administrative units where no DHS data exist. All correlations are significant at *P* = 0.001; parentheses show 95% CIs.

The better coverage of ML-based poverty maps does not come at the expense of accuracy. Rather, we find that the ML-based poverty maps measure the spatial distribution of poverty with approximately the same accuracy as the benchmark survey. This can be seen in Fig. 2*B*, which measures the accuracy of survey-based and ML-based poverty maps using a third independent source of ground truth data, Nigeria’s National Living Standards Survey (NLSS) of 22,110 households conducted in 2018 and 2019. At all levels of spatial disaggregation, the correlation with the NLSS is similar. Note that we do not expect the ML-based estimates to outperform the DHS-based estimates since the DHS data were used to train the ML-based model (*ML-Based Poverty Maps*). Rather, the main advantage of the ML-based maps is that they allow for accurate extrapolation of wealth estimates into the large number of regions not surveyed by the DHS.

We further find that correlations with ground truth for both DHS and ML-based poverty maps increase when we consider only the regions where the evaluation (NLSS) data are most reliable. (This analysis is intended to address one limitation of our empirical setting, noted in *Issues of Incomplete Survey Coverage*, which is that the ground-truth NLSS data used to evaluate performance are incomplete.) These results are shown in the last two columns of Table 1, which reports the correlation between the two poverty maps with ground truth estimates from the NLSS. While rows 1, 2, and 7 echo the results shown in Fig. 2*B*, the other rows indicate the correlation in specific subsets of the administrative units. In particular, we find that the performance of models evaluated using data from all LGAs (row 2) is inferior to that of models using only data from LGAs with at least 30 households in the NLSS (row 3). This effect is even stronger in Table 1, ward-level correlations, when we compare the analysis of all wards (row 7) with that of wards with at least 20 households (row 8). As expected, both DHS and ML-based poverty maps are more strongly correlated with the NLSS validation data when regions with the fewest households surveyed are excluded.

Perhaps most important, we find that the ML-based estimates remain accurate even when evaluated in regions where no DHS occurred. The accuracy of ML-based estimates in regions not covered by the DHS (but present in the NLSS ground truth and ML-based estimates) can be seen in rows 5 to 6 and 10 to 11 of Table 1. For instance, comparing rows 7 and 10, we see that the correlation between the ML-based estimates and ground truth is very similar (0.77 vs. 0.76). There is a slight attenuation in accuracy at the LGA level (row 2 vs. row 5), but this is likely due to the fact that the NLSS validation data are sparser in regions with no DHS data. Thus, we recalculate these correlations removing regions with little NLSS data (rows 6 and 11); the gap in accuracy shrinks at the LGA level (row 3 vs. row 6) and disappears at the ward level (row 8 vs. row 11). Overall, there is little evidence that the performance of ML-based maps deteriorates in regions where training data were unavailable.

### Results of National Targeting Simulations.

The third set of results, which is likely most relevant to policy makers, compares targeting outcomes using the ML-based wealth estimates with targeting outcomes using survey benchmark wealth estimates. The analysis is based on simulations of ward-level geographic targeting, where all households in selected wards receive an equal benefit and no households in unselected wards receive benefits. The data and methods used to construct poverty maps from the ML-based and survey-based data sources are described in *Primary Data Sources and Poverty Map Construction*. The details of the targeting simulations used to evaluate both methods are provided in *Targeting Simulations*.

To summarize, we find that, using a variety of different methods for evaluating targeting performance, the ML-based poverty maps would deliver a higher proportion of benefits to the poorest people in Nigeria than the survey-based benchmark (the DHS with representative imputation). This is true whether the goal of targeting is to provide benefits to the poor (defined as those consuming less than US $1.05 per day) or the extreme poor (consuming less than US $0.57 per day).

**Fig. 3.**
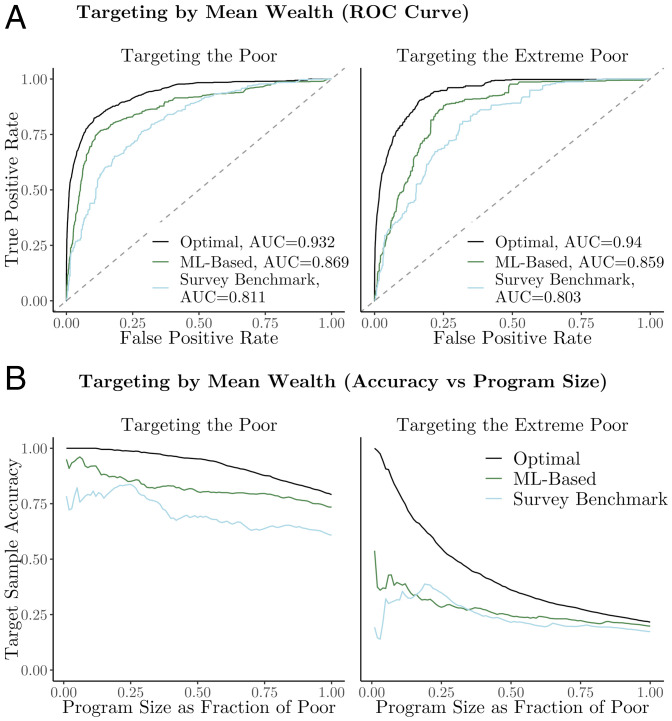
Ward-level targeting performance. Curves show performance of geographic targeting for programs of different sizes for three different approaches to targeting: an optimal approach based on the NLSS evaluation data; the ML-based approach based on high-resolution poverty maps; and a survey benchmark based on DHS data, imputing a representative portion of missing values. *A* shows ROC curves based on whether the NLSS households in targeted wards are poor (*Left*) or extreme poor (*Right*). *B* shows the fraction of program benefits going to the poor (*Left*) and extreme poor (*Right*) as the size of the antipoverty program varies.

The ROC curves in Fig. 3*A*, *Left* compare ward-level geographic targeting performance using the ML-based maps (area under the curve [AUC] = 0.87) with ward-level performance using the survey benchmark (AUC = 0.81). We also include, for reference, the performance of an “oracle” strategy (AUC = 0.93), which indicates the optimal performance that could be achieved with a purely geographic targeting approach. The survey benchmark shown in Fig. 3 imputes the wealth of a fraction of wards proportional to the number of wards with missing DHS data (“representative imputation” is discussed in *Issues of Incomplete Survey Coverage*) to ensure that every ward has a nonzero probability of receiving benefits. We separately measure the performance of a survey-based approach that is evaluated only in the 13.8% of wards with DHS data, which produces an AUC = 0.87. This approach performs similarly to the ML-based approach, but it could not be feasibly implemented because it would leave 86.2% of wards ineligible for benefits. Our findings are similar when we evaluate targeting based on the proportion of transfers reaching the extreme poor rather than the poor: ward-level targeting using the ML-based estimates (AUC = 0.86) improves on the survey benchmark (AUC = 0.80) and performs as well as the DHS when the DHS is only evaluated in the 13.8% of DHS wards (AUC = 0.86).

We find that ML-based maps can improve upon the survey-based benchmark for antipoverty programs of many sizes. Fig. 3*B* shows the fraction of transfers going to the poor and the extreme poor as the number of beneficiaries increases. We measure program size as a fraction of the total number of poor in Nigeria (currently estimated at 73.5 million). In Fig. 3*B*, *Left*, the ML-based map performs better than the benchmark irrespective of the size of the program. In Fig. 3*B*, *Right*, the ML-based map outperforms the benchmark for all extreme poverty program sizes, except those targeting a population of between 11.8 and 25.7 million people.

Comparing these two panels, it is evident that all targeting approaches perform better at targeting the poor than the extreme poor. This is because there are fewer extreme poor (8.2%) than poor households (40.5%), and the extreme poor are distributed throughout the country. Thus, any purely geographic targeting approach—no matter how accurate—will struggle to reach a large share of the extreme poor. This finding is consistent with past work on the difficulty of distinguishing poor and extreme poor households (14, 15).

To more concretely illustrate how the improvements in targeting accuracy from using the ML-based maps translate into better policy outcomes, Table 2 shows targeting precision at 10% recall (i.e., the fraction of transfers that reach the poor when 10% of the poor are targeted) (*SI Appendix*, Fig. S3 shows a visual comparison of the targeted regions). In Table 2, we observe a similar pattern as in Fig. 1: that targeting performance generally increases as smaller administrative units are targeted (i.e., when results in states are compared with LGAs and when LGAs are compared with wards).*

**Table 2.** Precision at 10% recall

	Precision at 10% recall		
Targeting approach	Targeting the poor	Targeting the extreme poor	Coverage, %
States			
Optimal (NLSS)	0.806	0.277	100
ML based	0.682	0.118	100
DHS based	0.795	0.218	100
LGAs			
Optimal (NLSS)	0.979	0.553	91.2
ML based	0.855	0.390	100
DHS based (representative imputation)	0.824	0.301	100
DHS based (minimum imputation)	0.843	0.321	91.2
DHS upper bound	0.840	0.359	81.5
Wards			
Optimal (NLSS)	1.000	0.976	22.9
ML based	0.919	0.406	100
DHS based (representative imputation)	0.793	0.311	100
DHS based (minimum imputation)	0.867	0.366	22.9
DHS upper bound	0.923	0.376	13.8

The first two columns indicate the fraction of transfers going to poor (or extreme poor) individuals when the program budget allows for 10% of the poor (or extreme poor) to be targeted. The third column indicates the proportion of each administrative unit for which the relevant dataset provides estimates (e.g., the NLSS conducted surveys in 91.2% of LGAs and 22.9% of wards). Optimal (NLSS) targeting uses the NLSS ground truth data to select the poorest administrative units for benefits. ML-based targeting selects units based on the average estimated wealth of those units. DHS-based targeting (representative imputation) selects units based on the average wealth of DHS households in that unit using an imputed wealth estimate on a representative fraction of units, reflecting the proportion of units in the country for which DHS estimates would have to be imputed. DHS-based targeting (minimum imputation) evaluates targeting performance in the full sample of NLSS wards, with missing wards imputed. DHS upper bound evaluates targeting performance only in units where the DHS occur.

Most important, we find that the ML-based approach outperforms the main DHS benchmark (representative imputation) at all levels of geographic targeting, except the state level (since the DHS is designed and weighted to be representative at the state level, it is expected to perform well—but state-level targeting would not be viable in Nigeria given the size of most states). In targeting the poor, the ML-based approach increases precision relative to the benchmark from 0.82 to 0.86 at the LGA level and from 0.79 to 0.92 at the ward level. In targeting the extreme poor, the increase is from 0.30 to 0.39 at the LGA level and from 0.31 to 0.41 at the ward level. At both the LGA and ward level, we see substantial overlap between regions selected by all three poverty maps (*SI Appendix*, Fig. S3).^†^

These increases in precision directly translate into reductions in errors of exclusion and inclusion. For instance, if we compare two geographically targeted antipoverty programs that each provide transfers to 7.3 million individuals (i.e., 10% of Nigeria’s poor population), the best ML-based approach (ward-level targeting) would correctly target 6,750,920 individuals; 66,735,620 poor individuals would not receive transfers, and 597,734 nonpoor individuals would be incorrectly included. DHS-based ward-level targeting would correctly target 5,787,802 individuals; 67,698,738 would be incorrectly excluded, and 1,560,852 would be incorrectly included. In other words, the ML-based approach would reduce exclusion errors by 1.4% and would reduce inclusion errors by 61.7%, resulting in nearly a million poor individuals receiving aid who otherwise would not have.

Our finding that ML-based targeting outperforms the survey benchmark is robust to several alternative approaches to targeting. Thus far, performance has been evaluated based on a method’s ability to target regions with low average (mean) wealth. When targeting is instead conducted based on median wealth, ML-based maps improve AUC over survey-based maps from 0.808 to 0.863 for targeting the poor and from 0.803 to 0.854 for targeting the extreme poor. Similar performance is observed for targeting based on the fraction of households in the ward that are (extreme) poor; AUCs for ML-based maps are 0.861 for targeting the poor vs. 0.802 for survey-based maps and 0.835 vs. 0.774 for targeting the extreme poor. *SI Appendix*, Fig. S6 has the corresponding ROC plots.

These results also remain qualitatively unchanged if we make different assumptions about the DHS benchmark. In particular, Table 2 also provides an upper-bound estimate of the performance of a survey benchmark by evaluating performance only in the 81.5% of LGAs and 13.8% of wards covered by the DHS. ML-based poverty maps outperform this DHS upper bound everywhere except when targeting the poor at the ward level, where the two approaches are nearly identical (precision of 0.923 vs. 0.919). We also include a “minimum imputation” estimate of DHS performance that is evaluated on the full NLSS sample (as opposed to the subset of NLSS regions that also appear in the DHS) by imputing DHS estimates for all regions where the DHS does not contain data (*SI Appendix*, *SI Text* and Fig. S8). The performance of minimum imputation typically exceeds that of representative imputation but falls below the DHS upper bound, and it always performs worse than the ML-based approach.

### Targeting Fairness and Demographic Parity.

Our final set of results explores the extent to which different targeting approaches (optimal [the NLSS], survey benchmark [the DHS; representative imputation], and ML-based) lead to a “fair” distribution of resources, where fairness is assessed based on statistical parity. This is motivated by the fact that a singular focus on the accuracy of targeting (at reaching the poor) might inadvertently concentrate benefits toward (or away from) specific, potentially marginalized or underserved, subgroups of the population (5, 6, 16–18). We note three results.

**Fig. 4.**
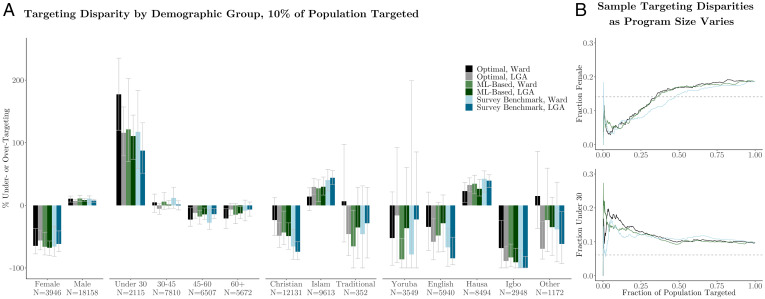
Comparison of targeting fairness for selected demographic groups (assigned based on the head of household). Under perfect individual targeting, the fraction of transfers going to members of a demographic group would be equal to the fraction of total poor households belonging to that demographic group. *A* shows the percentage difference between the number of households in each demographic group expected to receive transfers and the number that actually receive transfers, when 10% of the population is targeted. Error bars show bootstrapped 95% CIs. *B* shows how the fraction of transfers going to sample subgroups varies as a function of program size and as a fraction of total population. Dashed horizontal line indicates the proportion of poor households whose head of household belongs to that demographic group. Results pictured are for ward-level targeting.

First, geographic targeting can create demographic disparities—likely due to the fact that different subgroups of the population concentrate in specific geographic areas. These results can be seen in Fig. 4*A*, which quantifies the difference in the percentage of households of a certain group that are expected to receive transfers (based on the percentage of that group that is truly poor) and the percentage of households of that group that receive transfers according to each specific targeting method. In the figure, a large number of demographic subgroups (sets of bars) are statistically over- or undertargeted irrespective of the targeting methodology (indicated by bar color). For instance, even under optimal geographic targeting, Hausa speakers (40.0% of Nigerians per NLSS estimates) are overtargeted, and Igbo speakers (11.2% of Nigerians) are undertargeted. We also note significant undertargeting of female-headed households across all targeting strategies.

Second, spatial disaggregation has no clear effect on statistical disparities. With religion, we find that targeting smaller spatial units (i.e., wards) is marginally less disparate than targeting larger spatial units (i.e., LGAs). However, the opposite result appears when considering the age of the head of household. However, across all of these cases, confidence intervals (CIs, indicated by the whiskers) overlap. Thus, the overall impact of disaggregation may depend on the patterns of spatial heterogeneity in the specific regions under consideration.

Third and perhaps most relevant to the focus of this paper, we find that ML-based targeting leads to disparities that are similar in magnitude and direction to the survey-based benchmark. In Fig. 4*A*, we see that 95% CIs for ML- and survey-based targeting overlap significantly for all demographic groups. In Fig. 4*B*, we see similar results when the number of people targeted varies. While parity varies slightly for different program sizes, no systematic differences between targeting approaches are apparent.

## Discussion

This paper provides empirical evidence that recent advances in ML can improve the geographic targeting of social assistance. Our analysis, done to support the Government of Nigeria’s humanitarian response to the ongoing COVID-19 crisis, indicates that programs targeted using ML-based maps can direct more transfers to the poorest households than programs targeted using survey-based poverty maps. This improvement in targeting efficiency is due to the fact that the ML-based maps provide accurate estimates of the relative wealth of every administrative subdivision of the country, whereas survey data typically only cover a small fraction of all units. As a result, an ML-based approach can be designed for smaller regions, while a survey-based approach can only be designed for larger regions. We do not find evidence that ML-based poverty maps increase disparities between demographic groups in the Nigerian context.

### Limitations.

While promising, this approach and the results we document have important limitations. In particular, our analysis focuses on a comparison between targeting based on a large, standardized DHS (completed in 2018) and ML-based estimates trained on that survey, as evaluated using a more reliable NLSS with consumption data (completed in 2019). Some of our findings may be specific to this particular data environment. For instance, when the gap in time between the survey benchmark and the evaluation data is larger (or smaller), it is possible that the gains from ML-based estimates may differ. Our analysis in Nigeria also relies on ML-based estimates that are relatively accurate (ρ=0.802 for Admin-2 regions); when ML-based estimates are more (or less) accurate, we expect the relative benefits to increase (or decrease). Likewise, in countries with a different underlying wealth distribution or with different spatial patterns in the concentration of poverty, results may vary. Related, as discussed in *Ground Truth Evaluation Data from the NLSS* and *Issues of Incomplete Survey Coverage*, our reliance on the NLSS to evaluate both methods carries its own limitations; the NLSS surveys only a fraction of all geographic units and is not representative below the state level, and our evaluation relies on one particular asset-based notion of poverty, when in fact, poverty is complex and multidimensional. None of these limitations undermine the internal validity of our analysis, but they do highlight important avenues for future work.

Finally, we want to state clearly that this analysis should not be misconstrued to imply that ML-based approaches should replace survey-based methods for measuring poverty. Indeed, the ML-based approach was only feasible because high-quality survey data existed to train the ML model. More broadly, household surveys capture a wide range of information, with much greater nuance than can be clearly seen in overhead imagery and which may not be easily modeled with ML (19, 20). Rather, these results suggest that ML-based maps can provide a reliable method for geographic targeting when time and resource constraints prevent new data collection—a frequent consideration in the large number of LMICs without a recent census or comprehensive social registry.

## Materials and Methods

### Related Work.

This paper connects a recent strand of the applied ML literature to a rich literature in development economics. The most closely related ML papers explore how ML algorithms can be used to construct estimates of the spatial distribution of wealth and poverty from high-resolution satellite imagery (7–9, 20–24). Also related are papers that construct granular poverty maps from mobile phone (5, 25–28) and social media data (29) and recent work on evaluating fairness in ML-based targeting approaches (5, 6). Broadly speaking, these studies match nontraditional data (such as satellite or phone data) to a survey-based ground truth measure of wealth, train ML methods to predict wealth from the nontraditional data, and use the trained model to predict wealth estimates in regions where no ground truth data exist (30).

The second literature, which has a rich history in development economics, studies the targeting of social assistance and government transfers. This body of work provides theory and empirical evaluations of the different targeting mechanisms that are commonly used to determine eligibility for benefits (2, 3). The crux of the problem is that central governments often lack recent, reliable, and comprehensive data on the living conditions of each family (4). Thus, a variety of common targeting mechanisms exist to help direct benefits to the neediest households: self-targeting, where benefits are available to anyone, but there is some “ordeal” involved in registering such that only those with the greatest need will choose to participate (31, 32); proxy means tests, where wealth is estimated based on a small number of easily observed assets and housing characteristics (33, 34); community-based targeting, where communities are asked to identify their neediest members (35, 36); and geographic targeting, where resources are channeled to the regions with the highest levels of poverty (37–39). A key result from this literature, which we extend in this paper, is that significant efficiency gains can be achieved by targeting small administrative units rather than larger ones (11–13).

This paper connects these two historically disjoint literatures by examining whether recent innovations in ML-based poverty mapping can improve the targeting of social assistance and humanitarian aid (40). We build on prior work by Yeh et al. (8), who discuss the potential for ML-based maps in program targeting but stop short of analyzing a real-world policy decision and who do not compare the ML approach to status quo alternatives. We also build on recent work that shows how mobile phone metadata can improve targeting outcomes in Afghanistan (28) and Togo (5). Relative to these projects, our approach is likely most relevant in contexts where mobile phone data are not publicly available or when policy applications require a geographic approach to targeting.

### Targeting Context: Nigeria.

Our analysis was motivated by a specific request for assistance from the Government of Nigeria, which was working with the World Bank to design an emergency social assistance program in response to the COVID-19 crisis. At the onset of COVID-19, there was no single comprehensive social registry that would allow them to identify the individuals or households with the greatest need for assistance, and in the middle of the pandemic, it was impractical to go door to door to collect this information. Thus, they were interested in evaluating different approaches to geographic targeting.

Nigeria is home to roughly 211 million people, making it the seventh most populous country in the world. Geographically, Nigeria has three different levels of administrative subdivisions (Fig. 2); 37 states (Admin-1) are subdivided into a total of 774 LGAs (Admin-2), which in turn, are subdivided into a total of 8,808 wards (Admin-3). However, in early 2020, the best poverty data available to the Government of Nigeria could only provide estimates of state-level poverty; they did not allow for estimates of rates of poverty at the LGA or ward level.

Based in part on the analysis described in this paper, the government elected to use our high-resolution poverty maps to target the COVID-19 Rapid Response Registration (RRR) Cash Transfer Project, which began disbursing benefits to the first of an eventual 1 million recipients in mid-January 2021 (10). The RRR program is designed specifically to help the urban poor; *SI Appendix* has a separate evaluation of targeting outcomes for urban areas only.

### Primary Data Sources and Poverty Map Construction.

#### ML-based poverty maps.

The high-resolution poverty maps shown in Fig. 2*A*, row D are constructed using an ML approach described in greater detail in Chi et al. (9), which follows an approach similar to that first proposed by Jean et al. (7). To summarize, ground truth survey data from several DHSs provide information on the wealth of millions of households in LMICs, including 40,427 households across Nigeria. These “labels” are matched, using geographic markers in the survey dataset, to a rich set of nontraditional geospatial data, including features derived from high-resolution satellite imagery using a convolutional neural network as well as mobile connectivity data and other topological data. A gradient-boosted decision tree is then used to predict the labels from the satellite and other geospatial features using spatially stratified cross-validation. The fitted model is then used to predict the wealth of every 2.4-km gridded region in the country of Nigeria.

To produce estimates of the wealth and poverty of the different administrative units of Nigeria (Fig. 2*A*, *Right*, rows A–C), the 2.4-km estimates are aggregated using population weights, where the population of each 2.4-km grid cell is generated using population estimates from Humanitarian Data Exchange (41). Specifically, the wealth estimate of administrative unit *i* is calculated as

[1] $$W_{i}=\left(\frac{1}{\sum_{t\in T}I(t,i)p_{t}}\right)\sum_{t\in T}I(t,i)p_{t}w_{t},$$

where *T* is the set of all 2.4-km satellite tiles; *p* and *w* approximate the population and wealth of tile *t*, respectively; and *I* gives the fraction of tile *t* that intersects administrative unit *i*. Because wealth indices are relative and have no meaningful units, they are normalized at the administrative level to have a mean of zero and SD of one.

#### Benchmark poverty maps from the DHS.

As a benchmark against which we compare the targeting outcomes of the ML-based maps, we construct a set of poverty maps using data from a recent, nationally representative household survey. Specifically, we obtain the microdata from Nigeria’s 2018 DHS (42). The DHS is a standardized household survey funded by the US Agency for International Development; the 2018 Nigerian DHS conducted surveys with 40,427 households in 1,360 unique locations across the country. The survey instrument contains detailed questions about the socioeconomic conditions of each household, including a wealth index, which provides a scalar measure of the wealth of that household relative to all other surveyed households.^‡^ We also observe the approximate location of each DHS household, where the DHS groups households into clusters (roughly equivalent to villages in rural areas and neighborhoods in urban areas) and provides the geocoordinates of the centroid of the cluster of households after adding up to 5 km of jitter to preserve the privacy of individual households.

To construct poverty maps from the household survey data (as shown in Fig. 2*A*, *Left*), we calculate the average wealth index of all surveyed households located in the relevant administrative unit. For this process, we obtained shape files and urban/rural classifications for each administrative unit from the World Bank. Both the NLSS and the DHS were designed to provide estimates of population characteristics that are representative at the state level, and each household has an associated survey sampling weight. Thus, for the state-level poverty maps, we use this sampling weight to calculate the weighted average wealth index of all households in the state. When constructing LGA and ward wealth estimates, we take the simple average of all households in the relevant administrative unit since the household survey weights were not intended to provide LGA- or ward-representative inferences.

#### Ground truth evaluation data from the NLSS.

To evaluate the performance of targeting using the ML-based poverty maps and the survey-based poverty maps, we obtain a separate independent source of “ground truth” data on living standards in Nigeria. This is the 2019 NLSS, an ambitious household survey financed by the World Bank and implemented by Nigeria’s National Bureau of Statistics (43). The survey was conducted with 22,110 households, of which 22,104 had precise geo-coordinates. For each household in this dataset, we observe the exact geocoordinates as well as a rich set of questions about socioeconomic conditions. We use the responses to these questions to construct a DHS-style wealth index for each NLSS household.^§^ The NLSS was never used to train the ML-based poverty maps and did not influence the collection of the DHS data; it thus provides an objective and out-of-sample means for validating the alternative approaches to geographic targeting. *SI Appendix* has a detailed description of the survey methodology and data availability.

#### Data limitations.

In important ways, our analysis is constrained by the incompleteness of ground truth data, a point discussed in detail in *Issues of Incomplete Survey Coverage*. To summarize, while the scope and scale of the NLSS were quite extensive relative to survey efforts in many LMICs, it still did not collect data in 8.8% of LGAs and 77.1% of wards. Since the NLSS was carefully designed to be nationally representative, our main analysis assumes that evaluation performed on the NLSS sample will generalize to the rest of the country. We cannot test this assumption directly since we do not have evaluation data beyond what is available in the NLSS. However, our analysis in Table 1 and *SI Appendix*, Table S1 tests this indirectly by assessing the accuracy of ML-based maps in regions with and without DHS data. Since the DHS was also designed to be nationally representative, this analysis may shed light on the limits of evaluating performance on incomplete but nationally representative survey data. In Table 1, we observe that the performance of the ML-based maps does not depend critically on the availability of DHS data. However, in *SI Appendix*, Table S1, we do observe a decrease in performance in very remote regions (*SI Appendix*, *Accuracy of Targeting Data in Remote Regions* has details).

A second limitation of the NLSS is that it was designed to be representative at the state level, meaning that households sampled at the smaller LGA and ward levels are not guaranteed to be representative of their administrative units. We test the importance of this limitation in Table 1 by assessing performance in regions with more and fewer surveyed households, as we expect the sparser regions to have the highest variance (Table 1). As expected, the accuracy of both the ML-based and the survey-based maps is higher in regions with more surveyed households. However, we do not see evidence suggesting that our estimate of the relative performance of ML-based and survey-based maps is meaningfully changed by this source of measurement error in the NLSS data.

### Targeting Simulations.

We simulate the geographic targeting of antipoverty programs in Nigeria using two different approaches—one based on the ML-based poverty maps (derived from satellite imagery) and the other based on the survey-based benchmark (derived from the 2018 DHS). The performance of these two approaches is evaluated using ground truth data derived from the 2018 to 2019 NLSS, which is considered the most comprehensive and up-to-date survey in Nigeria.

Specifically, we assess targeting performance based on the proportion of transfers that would reach poor and extreme poor households under different approaches to geographic targeting. Using the Nigeria-specific World Bank poverty line of 377 Nigerian Naira per person per day ($1.05 in 2018), we estimate from the NLSS data that 40.5% of the population is poor (consumption below the poverty line) and that 8.2% of the population is extremely poor (consumption below half the poverty line). Since neither the DHS nor the ML model provide direct income or consumption data, our simulations of DHS and ML-based targeting focus on the 40.5% and 8.2% of households with the lowest wealth. Thus, households with wealth indices in the bottom 8.2% are classified as extreme poor, and those in the bottom 40.5% are classified as poor (i.e., poor is inclusive of extreme poor).

Based on these thresholds, we can classify each household in the NLSS evaluation data as extreme poor, poor, and nonpoor; we can likewise calculate the fraction of households in each administrative unit that falls into each category of poverty. When calculating state-level poverty rates, we use the survey sample weights; no weights are used to calculate poverty rates at the LGA and ward levels.

We then simulate geographic targeting policies at the state, LGA, and ward levels, where the targeting is determined using estimates from the ML-based poverty map (“ML-based method”), the DHS-based poverty map data (the “benchmark method”), and the NLSS-based poverty map (the “oracle method”). Under each approach, we assume that 100% of the households within a given administrative unit will receive the same benefit, which is how the Nigerian government originally envisioned this program would be implemented. Note that this implies that even the oracle method, where geographic targeting is determined by the same dataset used to evaluate targeting, will not be perfectly accurate. This is because there exist nonpoor households in even the poorest wards of Nigeria, so providing benefits to everyone in the poorest wards will result in errors of inclusion. Likewise, errors of exclusion will occur whenever poor individuals live in wealthy regions—even if the targeting data can perfectly separate wealthy from poor regions.

While ward-level targeting theoretically has a higher upper bound on performance, estimates at the LGA and state levels can draw on more data and thus, may be more accurate. It is useful to analyze targeting performance for these administrative units as well to quantify the trade-off between greater targeting precision (at the ward level) and potentially more accurate wealth estimates (at the LGA/state level).

#### Alternative targeting criteria.

In addition to the poverty maps used in our main specification, which estimate mean poverty of each administrative unit, we create two additional ward-level poverty maps from each data source as a robustness check. The first estimates median poverty. Because the NLSS and DHS sample weights are not representative at the ward level, we use the poverty level of the unweighted median household in each ward. Median wealth is calculated from the ML-based map using the median of the wealth estimates of each 2.4-km satellite tile, weighted by the estimated population in that tile. The second additional map estimates poverty rate and extreme poverty rate of each ward. To estimate these poverty rates, NLSS and DHS households are classified as (extreme) poor based on the percentile of their wealth index (*Targeting Simulations*). The unweighted fraction of households in each ward that are (extreme) poor is used as the targeting criteria for the NLSS and the DHS. For the ML-based map, each 2.4-km satellite tile is classified as (extreme) poor based on the percentile of its estimated wealth index, the fraction of people in each ward who are (extreme) poor is then calculated as the fraction of people who live in satellite tiles that are classified as (extreme) poor. Since separate maps are created for the poor and extreme poor, the order in which wards are targeted depends on which map is being used for targeting.

### Issues of Incomplete Survey Coverage.

One limitation of the surveys—both the DHS data used to construct benchmark estimates of poverty and the NLSS data used to evaluate targeting performance—is that the data are sparse. As we discuss in greater detail below, only 13.8% of Nigerian wards have one or more surveyed DHS households, and only 22.9% of wards have one or more households in the NLSS.

#### Incomplete evaluation data.

While great care was taken in the design of the NLSS to ensure that the survey population was representative of the full population of Nigeria (and also representative of each state), we are unable to evaluate the performance of the ML-based and survey-based poverty maps in the 8.8% of LGAs and 77.1% of wards where no ground truth NLSS data exist. To ensure that results are comparable for all targeting approaches, we further limit our results on targeting accuracy to the 77% of LGAs and 5% of wards where both the NLSS and the DHS include at least one household. When evaluating targeting for LGAs and wards, we also report results when performance is measured only on the subset of wards where the NLSS contains at least 20 households and the subset of LGAs where the NLSS contains at least 30 households. This effectively removes the wards and LGAs where our ground truth estimates of poverty have the highest variance.

#### Incomplete benchmark data.

The fact that the DHS data are only collected in a small fraction of all geographic units of the country means that those data cannot be used in isolation to determine a ward- or LGA-level geographic targeting policy. Instead, either the policy would need to be designed at the state level (where DHS coverage is complete), or some form of imputation would be required to make decisions about LGAs and wards where data do not exist.

In the targeting simulations, we simulate the performance of geographic targeting with DHS data using three different methods. The first, “DHS upper bound,” considers only those administrative units where both the DHS and the NLSS were conducted (5.3% of all wards and 76.9% of all LGAs). This approach requires no imputation and provides an indication of the performance to be expected if it was possible to collect DHS data in 100% of wards. The resources required for such a data collection effort make this approach impractical, but it represents an upper bound on the performance of targeting with DHS data.

The second method, “DHS based (minimum imputation),” considers all 706 LGAs and 2,016 wards covered by the NLSS—even though many of those units do not have any households covered by the DHS. Specifically, in the 597 LGAs and 464 wards where DHS data are available, DHS estimates are used directly. In the remaining 109 LGAs and 1,552 wards, wealth estimates are imputed as the average (population-weighted) wealth of the larger spatial unit in which the smaller unit falls (i.e., we use the average LGA wealth for wards with no DHS and the average state wealth for LGAs with no surveys).

The third method, “DHS based (representative imputation),” simulates what we believe is the most realistic scenario by imputing data for a fraction of wards and LGAs to match the true missingness in the national data. To ensure comparability with the DHS upper-bound method, our primary results use the matched sample of 597 LGAs and 464 wards where both the DHS and the NLSS are available. Using the minimum imputation method, we also show results for the larger sample of regions where NLSS data are available in *SI Appendix*, Fig. S8.

Specifically, we impute the wealth $W_{\text{L}}(i)$ of LGA *i* for a randomly selected subset of LGAs as

[2] $$\begin{array}{r} {\hat{W}}_{\text{L}}(i)=\left(\frac{1}{A_{i,\text{L}}}\right)\sum_{h\in H}\left(a_{h}w_{h}\right)1\{\text{S}(h)=\text{S}(i),\;\text{L}(h)\neq i\}\\ A_{i,\text{L}}=\sum_{h\in H}a_{h}1\{\text{S}(h)=\text{S}(i),\;\text{L}(h)\neq i\}. \end{array}$$

Here, *H* is the set of all surveyed households, and *h* is a household in state S(*h*) and LGA L(*h*), with survey weight *a_h_* and wealth index *w_h_*. Intuitively, this gives the survey-weighted mean wealth of households in the same state as a given LGA but not within the LGA itself. We similarly impute the wealth ${\hat{W}}_{\text{W}}(i)$ of ward *i* for a randomly selected subset of wards as

[3] $${\hat{W}}_{\text{W}}(i)=\{\begin{array}{ll} \left(\frac{1}{A_{i,\text{W}}}\right)\sum_{h\in H}w_{h}1\{\text{L}(h)=\text{L}(i),\text{W}(h)\neq i\} & \exists h\in H_{i,\text{W}}\\ {\hat{W}}_{\text{L}}(L(i)) & ∄h\in H_{i,\text{W}}, \end{array}$$

where $A_{i,\text{W}}$ is defined analogously to Eq. **2** and $H_{i,\text{W}}$ is the set of all households in the same LGA as ward *i* but not in ward *i*. W(*h*) gives the ward in which survey household *h* is located. Thus, if at least one household exists in the survey data that is in the same LGA as a given ward, the ward is imputed as the simple average of all households in the LGA. Otherwise, the ward’s wealth is imputed as the survey-weighted average of all households in the same state as the ward but not the ward itself.

Our goal with this third method is to make the imputation representative of the true missingness in the DHS. Thus, we randomly replace 18.5% of LGAs and 86.2% of wards with imputed values since 18.5% of LGAs and 86.2% of wards do not have the DHS (and would thus require imputed estimates in practice). We repeat this randomization process 1,000 times and report results for the iteration with the median performance, as determined by the area under the ROC curve.

### Estimating Demographic Parity.

We estimate the “fairness” of targeting based on statistical parity (16), which defines a fair allocation as one in which the fraction of households in a specific group receiving transfers is equal to the fraction of households in that group that are truly poor. We acknowledge that other notions of fairness exist and may conflict with this focus on statistical parity (45).

Our analysis assesses statistical parity for four demographic characteristics that are recorded in the NLSS: gender, age, religion, and language (a proxy for ethnicity, which is not recorded). We observe these characteristics just for the head of household, so our evaluation focuses on the extent to which households with a household head of a certain type are under- or overtargeted.

The fractions of households in each ward in each demographic category are estimated using NLSS data. NLSS data are also used to estimate the total fraction of poor households that belong to each demographic group. This reference statistic is calculated for the subset of wards in which targeting simulations occur (i.e., those with coverage in both the NLSS and the DHS); thus, it may not accurately reflect the country-level demographics of poor households.

For each of the three targeting approaches –optimal (the NLSS), survey benchmark (the DHS with representative imputation) and ML-based –we calculate the fraction of targeted households that belong to each demographic group. These fractions are evaluated using ward-level demographic information from the NLSS, and vary based on the number of targeted households. We also calculate a snapshot of parity for a program targeting 10% of the population. For demographic group *d*, we calculate the extent of over- or undertargeting using

[4] $$\frac{\text{Fraction}\;\text{of}\;\text{targeted}\;\text{in}\;d-\text{Fraction}\;\text{of}\;\text{poor}\;\text{in}\;d}{\text{Fraction}\;\text{of}\;\text{poor}\;\text{in}\;d}\cdot 100.$$

We generate CIs by bootstrap sampling Eq. **4** 1,000 times.

## Supplementary Material

Supplementary File

## Data Availability

The ML-based estimates of wealth were contsructed by Chi et al. (9) and are available from the Humanitarian Data Exchange (https://data.humdata.org/dataset/relative-wealth-index). Population estimates are publicly available from the High Resolution Settlement Layer (41). DHS data survey data are available upon registration at https://www.dhsprogram.com/ (42). NLSS survey data must be requested from the Nigerian National Bureau of Statistics (43). The code used for these analyses, along with direct links to required data sources, is available at https://github.com/issmythe/nigeria_poverty_mapping (46).

## Footnotes

^*^Note that while targeting performance increases with spatial disaggregation in Table 2, we earlier saw in Table 1 that the correlation between the NLSS ground truth and both the ML-based and DHS-based poverty maps decreased with spatial disaggregation. This illustrates a bias–variance trade-off, where the smaller units of analysis imply that fewer households are available to calculate the average wealth of a region.

^†^The analysis in Table 2 is based on wards where both the DHS and the NLSS contain at least one household (*N* = 464). There is also a large number of wards for which the DHS does not contain data, but the NLSS does (*N* = 1,552). In these regions, it is possible to evaluate ML-based targeting performance but not the survey-based benchmark. We focus on wards where all data are available to facilitate direct comparisons between the DHS and ML model. In an analysis not reported in Table 2, we find that the targeting accuracy of the ML-based approach is largely unchanged when evaluated on the full set of 1,552 wards with NLSS data. For targeting the poor, AUC remains virtually unchanged (0.867 vs. 0.869 for DHS wards only). For targeting the extreme poor, we observe a slightly larger decline (to 0.82 from 0.86). Precision at 10% recall is unchanged at 0.92 for targeting the poor and declines slightly from 0.41 to 0.39 for targeting the extreme poor (*SI Appendix*, *SI Text* and Fig. S8).

^‡^The wealth index is constructed as the first principal component of a vector of assets and household characteristics: air conditioner, animal-drawn cart, bank account, bed, bicycle, boat with a motor, canoe, car or truck, chair, computer, cupboard, electric iron, electricity, fan, generator, landline, motorbike, main floor material, main roof material, main wall material, mobile telephone, motorcycle or scooter, number of members per sleeping room, owns a house, owns land, radio, refrigerator, sofa, source of drinking water, table, television, type of toilet facility, type of cooking fuel, and watch.

^§^The NLSS is more detailed than the DHS and contains a superset of the DHS asset questions. We, therefore, use the principal component analysis weights from the DHS wealth index to calculate the wealth index of each NLSS household (rather than calculating a new set of eigenvectors from the NLSS data).

## References

[1] U. Gentilini, M. Almenfi, I. Orton, P. Dale, Social protection and jobs responses to COVID-19. World Bank, Washington, DC (2021). https://openknowledge.worldbank.org/handle/10986/33635. Accessed 1 September 2021.

[2] D. Coady, M. Grosh, J. Hoddinott, Targeting outcomes redux. World Bank Res. Obs. 19, 61–85 (2004).

[3] R. Hanna, B. A. Olken, Universal basic incomes versus targeted transfers: Anti-poverty programs in developing countries. J. Econ. Perspect. 32, 201–226 (2018).

[4] M. Jerven, Poor Numbers: How We Are Misled by African Development Statistics and What to Do about It (Cornell University Press, 2013).

[5] E. Aiken, S. Bellue, D. Karlan, C. Udry, J. E. Blumenstock, Machine learning and phone data can improve targeting of humanitarian aid. Nature 603, 864–870 (2022). 35296856 10.1038/s41586-022-04484-9 PMC8967719

[6] L. Kondmann, X. X. Zhu, Under the radar—auditing fairness in ML for humanitarian mapping. arXiv [Preprint] (2021). https://arxiv.org/abs/2108.02137 (Accessed 1 September 2021).

[7] N. Jean ., Combining satellite imagery and machine learning to predict poverty. Science 353, 790–794 (2016). 27540167 10.1126/science.aaf7894

[8] C. Yeh ., Using publicly available satellite imagery and deep learning to understand economic well-being in Africa. Nat. Commun. 11, 2583 (2020). 32444658 10.1038/s41467-020-16185-w PMC7244551

[9] G. Chi, H. Fang, S. Chatterjee, J. E. Blumenstock, Microestimates of wealth for all low- and middle-income countries. Proc. Natl. Acad. Sci. 119, e2113658119 (2022). 35017299 10.1073/pnas.2113658119 PMC8784134

[10] News Agency of Nigeria, 1 million Nigerians to benefit from COVID-19 cash transfer programme. *Pulse Niger*, 19 January 2021. https://www.pulse.ng/news/local/osinbajo-1-million-nigerians-benefit-from-covid-19-cash-transfer/jf73y7s. Accessed 1 February 2021.

[11] M. Ravallion, “Poverty alleviation through regional targeting: A case study for Indonesia” in The Economics of Rural Organization: Theory, Practice and Policy, K. Hoff, A. Braverman, J. E. Stiglitz, Eds. 373–377 (Oxford University Press, New York, NY, 1993).

[12] D. P. Coady, The welfare returns to finer targeting: The case of the progresa program in Mexico. Int. Tax Public Finance 13, 217–239 (2006).

[13] C. Elbers, T. Fujii, P. Lanjouw, B. Özler, W. Yin, Poverty alleviation through geographic targeting: How much does disaggregation help? J. Dev. Econ. 83, 198–213 (2007).

[14] C. Brown, M. Ravallion, D. Van de Walle, A poor means test? Econometric targeting in Africa. J. Dev. Econ. 134, 109–124 (2018).

[15] H. A. Dang, D. Jolliffe, C. Carletto, Data gaps, data incomparability, and data imputation: A review of poverty measurement methods for data-scarce environments. J. Econ. Surv. 33, 757–797 (2019).

[16] C. Dwork, M. Hardt, T. Pitassi, O. Reingold, R. Zemel, “Fairness through awareness” in *Proceedings of the 3rd Innovations in Theoretical Computer Science Conference* (2012), pp. 214–226 (Association for Computing Machinery, New York, NY, United States).

[17] A. Noriega-Campero ., “Algorithmic targeting of social policies: Fairness, accuracy, and distributed governance” in *Proceedings of the 2020 Conference on Fairness, Accountability, and Transparency* (Association for Computing Machinery, New York, NY, 2020), pp. 241–251.

[18] S. Barocas, M. Hardt, A. Narayanan, Fairness and machine learning: Limitations and opportunities (2019). www.fairmlbook.org. Accessed 1 September 2021.

[19] J. Blumenstock, Don’t forget people in the use of big data for development. Nature 561, 170–172 (2018). 30202055 10.1038/d41586-018-06215-5

[20] A. Head, M. Manguin, N. Tran, J. E. Blumenstock, “Can human development be measured with satellite imagery?” in *Proceedings of the Ninth ACM/IEEE International Conference on Information and Communication Technologies and Development (ICTD 2017)* (ACM, Lahore, Pakistan, 2017) pp. 16–19.

[21] B. Babenko, J. Hersh, D. Newhouse, A. Ramakrishnan, T. Swartz, Poverty mapping using convolutional neural networks trained on high and medium resolution satellite images, with an application in Mexico. arXiv [Preprint] (2017). https://arxiv.org/abs/1711.06323 (Accessed 1 December 2020).

[22] R. Engstrom, J. Hersh, D. Newhouse, Poverty from space: Using high-resolution satellite imagery for estimating economic well-being (Policy Research Working Paper 8284, World Bank, 2017, http://hdl.handle.net/10986/29075).

[23] J. L. Abitbol, M. Karsai, Interpretable socioeconomic status inference from aerial imagery through urban patterns. Nat. Mach. Intell. 2, 684–692 (2020).

[24] W. Nordhaus, X. Chen, A sharper image? Estimates of the precision of nighttime lights as a proxy for economic statistics. J. Econ. Geogr. 15, 217–246 (2015).

[25] J. Blumenstock, G. Cadamuro, R. On, Predicting poverty and wealth from mobile phone metadata. Science 350, 1073–1076 (2015). 26612950 10.1126/science.aac4420

[26] J. E. Steele ., Mapping poverty using mobile phone and satellite data. J. R. Soc. Interface 14, 20160690 (2017). 28148765 10.1098/rsif.2016.0690 PMC5332562

[27] J. E. Blumenstock, Estimating economic characteristics with phone data. Am. Econ. Rev. Pap. Proc 108, 72–76 (2018).

[28] E. Aiken, G. Bedoya, A. Coville, J. E. Blumenstock, Program targeting with machine learning and mobile phone data: Evidence from an anti-poverty intervention in Afghanistan. arXiv [Preprint] (2022). https://arxiv.org/abs/2206.11400?context=q-fin.EC (Accessed 22 June 2022).

[29] M. Fatehkia ., Mapping socioeconomic indicators using social media advertising data. EPJ Data Sci. 9, 22 (2020).

[30] J. E. Blumenstock, Fighting poverty with data. Science 353, 753–754 (2016). 27540154 10.1126/science.aah5217

[31] D. Nichols, E. Smolensky, T. N. Tideman, Discrimination by waiting time in merit goods. Am. Econ. Rev. 61, 312–323 (1971).

[32] V. Alatas ., Self-targeting: Evidence from a field experiment in Indonesia. J. Polit. Econ. 124, 371–427 (2016).

[33] M. Grosh, J. L. Baker, Proxy means tests for targeting social programs. Living standards measurement study working paper 118, 1–49 (1995). 10.1596/0-8213-3313-5. Accessed 1 December 2020.

[34] D. Filmer, L. H. Pritchett, Estimating wealth effects without expenditure data–or tears: An application to educational enrollments in states of India. Demography 38, 115–132 (2001). 11227840 10.1353/dem.2001.0003

[35] H. Alderman, Do local officials know something we don’t? Decentralization of targeted transfers in Albania. J. Public Econ. 83, 375–404 (2002).

[36] V. Alatas, A. Banerjee, R. Hanna, B. A. Olken, J. Tobias, Targeting the poor: Evidence from a field experiment in Indonesia. Am. Econ. Rev. 102, 1206–1240 (2012). 25197099 10.1257/aer.102.4.1206 PMC4156293

[37] J. L. Baker, M. E. Grosh, Poverty reduction through geographic targeting: How well does it work? World Dev. 22, 983–995 (1994).

[38] N. R. Schady, Picking the poor: Indicators for geographic targeting in Peru. Rev. Income Wealth 48, 417–433 (2002).

[39] T. Bedi, A. Coudouel, K. Simler, More than a Pretty Picture: Using Poverty Maps to Design Better Policies and Interventions (World Bank Publications, 2007).

[40] J. Blumenstock, Machine learning can help get COVID-19 aid to those who need it most. Nature, 10.1038/d41586-020-01393-7 (2020). 32409767

[41] Facebook Connectivity Lab and Center for International Earth Science Information Network - CIESIN - Columbia University. High Resolution Settlement Layer (HRSL) (2016). Source imagery for HRSL [copyright] 2016 DigitalGlobe. https://ciesin.columbia.edu/data/hrsl/. Accessed 1 June 2020.

[42] National Population Commission; ICF, “National Population Commission - NPC, ICF, Nigeria Demographic and Health Survey 2018” (National Population Commission and ICF, Abuja, Nigeria, 2019).

[43] National Bureau of Statistics, Federal Government of Nigeria, “Nigeria living standards survey 2018-2019” (National Bureau of Statistics, Federal Government of Nigeria, Abuja, Nigeria, 2020).

[44] A. Deaton, “Measuring poverty” in Understanding Poverty, A. V. Banerjee, R. Bénabou, D. Mookherjee, Eds. (Oxford University Press, Oxford, United Kingdom, 2006), pp. 3–15.

[45] J. Kleinberg, S. Mullainathan, M. Raghavan, Inherent trade-offs in the fair determination of risk scores. arXiv [Preprint] (2016). https://arxiv.org/abs/1609.05807 (Accessed 1 September 2021).

[46] I. S. Smythe, nigeria_poverty_mapping. GitHub. https://github.com/issmythe/nigeria_poverty_mapping. Deposited 12 July 2022.

