# Supplementary material for: Geographic microtargeting of social assistance with high-resolution poverty maps
Source: Proc Natl Acad Sci U S A. 2022 Aug 1;119(32):e2120025119. doi: 10.1073/pnas.2120025119 (PMC9371736; doi:10.1073/pnas.2120025119)
Supplement: Supplementary File [file pnas.2120025119.sapp.pdf]

Supplementary Information for

Geographic Micro-Targeting of Social Assistance with High-Resolution Poverty Maps

Isabella Smythe and Joshua E. Blumenstock

Joshua E. Blumenstock
E-mail: jblumenstock@berkeley.edu

This PDF file includes:

Supplementary text
Figs. S1 to S10 (not allowed for Brief Reports)
Table S1 (not allowed for Brief Reports)
SI References

Supporting Information Text

Nigerian Living Standards Survey

The Nigerian Living Standards Survey (NLSS) is a nationally representative survey conducted by the Nigerian National Bureau of Statistics (NBS) in partnership with the World Bank. It contains data for 22,110 households, 22,104 with exact locations recorded, collected between September of 2018 and September of 2019. The NLSS sample frame is designed to be representative at the state level. The sample frame is based on the Nigeria Integrated Survey of Households 2 (NISH2), a national master sample developed by the NBS and based on enumeration areas (EAs) from the 2006 census.

Two questionnaires were administered to each surveyed household. The first collected household-level data, including demographics; assets, expenditures, and housing characteristics; and education and sources of income. The second collected community-level data, including local prices and infrastructure.

Ten households were surveyed per selected enumeration area (EA). A small number of EAs (6%) could not be accessed for security reasons, and were replaced with alternate EAs. The NLSS is not representative for Borno state, where ongoing conflict disrupted data collection. Samples were weighted and calibrated at the state level to match estimated 2019 populations.

The NLSS microdata are available from the National Bureau of Statistics of Nigeria*. The NBS project page also provides comprehensive documentation on the NLSS survey design.

Targeting urban wards

We also evaluate targeting performance in only urban wards, where a ward's status as "urban" is determined by the National Bureau of Statistics of Nigeria. We include these results because the eventual decision made by policymakers in Nigeria was to focus a geographic targeting program on urban wards (1), and this analysis proved instrumental to the decision to rely on ML-based poverty maps.

In urban areas, the ML-based maps are correlated nearly as tightly ($\rho = 0.74$) with the NLSS-based ground truth estimates as the DHS-based maps ($\rho = 0.75$). We also note that the correlations in urban wards ($0.74 \leq \rho \leq 0.75$) are quite similar to those of all wards ($0.77 \leq \rho \leq 0.78$). This similarity is reassuring since we would generally expect that the set of urban wards are more homogeneous (and therefore harder to differentiate using satellite imagery) than the set of all wards.

Urban targeting outcomes are also better under ML-based geographic targeting than benchmark DHS geographic targeting. This can be seen in the ROC curves of Figure S5 Panel A, where the ML-based maps consistently outperform the DHS approach in targeting the extreme poor, and achieve a higher AUC in targeting the poor. We note that the ROC curve for the ML approach is slightly inferior to the DHS benchmark in wealthier wards (a pattern that did not appear in the full-country results). However, Figure S5 Panel A shows that the overall targeting performance of the ML-based approach still dominates the benchmark for nearly all program sizes up to 100% of the total population of urban poor.

Correlations between imputed DHS wealth estimates and ground truth

Targeting simulations for the survey benchmark poverty map are conducted by replacing a portion of true DHS wealth estimates with imputed wealth estimates. Imputation is done using DHS data from nearby regions; see Section E.2 for details. Figure S7 plots these imputed wealth estimates against NLSS ground truth wealth estimates for the corresponding regions. As expected, the correlations between imputed DHS estimates and ground truth wealth are lower than the original DHS estimates (correlation declines from 0.787 to 0.647 for LGAs, and from 0.779 to 0.663 for wards). This reflects a decline in accuracy of DHS wealth estimates in regions where data is unavailable.

Targeting all wards in the ground truth sample

Our main analysis compares the performance of targeting using optimal, ML-based, and survey benchmark poverty maps on the matched sample of 5.3% of wards where both the optimal (NLSS) and benchmark (DHS) surveys have coverage. It is also possible to evaluate performance on the full 22.9% of wards for which NLSS (but not necessarily DHS) data are available by using the process described in Section E.2 to impute the missing wealth of DHS wards where no DHS data exist ("minimum imputation DHS"). Of the 2,016 wards that have NLSS data (22.9% of all wards in Nigeria), the DHS contains data for 464 wards (23.0% of the 22.9%); the wealth estimates for the remaining 1,552 wards are imputed.

For targeting the poor (Figure S8 Panel A), ML-based targeting is virtually unchanged (AUC shifts from 0.869 to 0.867), while the survey benchmark targeting improves slightly (from 0.811 to 0.842). For targeting the extreme poor, the ML-based targeting AUC declines slightly from 0.859 to 0.822, while the survey benchmark AUC is unchanged (from 0.803 to 0.807).

It is reassuring that the performance of the ML-based approach is nearly the same in this sample, as it suggests that missingness of validation data was not biasing our estimates. The modest increase that we observe in the performance of DHS-based ward-level targeting is expected, since it uses DHS survey data directly for 23.0% of wards, and imputed estimates for 77.0%; by contrast, the original results in Figure 3 use DHS data directly in 13.8% of wards, and use imputed estimates for 86.2%.

* https://nigerianstat.gov.ng/nada/index.php/catalog/68/study-description

Accuracy of targeting data in remote regions

One of the advantages of ML-based maps is that they make it possible to geographically target in regions where no surveys exist. The analysis in Table 1 suggests that correlations are comparable in regions with and without DHS data. In Table S1, we evaluate the performance of ML-based maps in more remote regions, by assessing how the correlation between the ML-based estimates and the NLSS ground truth changes based on the availability of DHS training data.

This table suggests a modest decline in the accuracy of the ML-based maps in areas that are very far from locations with DHS training data (column 2). However, there are relatively few people living in wards that are very far from DHS clusters (column 1). For instance, 81.4% of wards (containing 86.8% of the population) are within 10 kilometers of the nearest DHS cluster. If we exclude Borno, where the most recent DHS survey was not completed because of an ongoing conflict, the percentages increase to 83.4% of wards comprising 88.2% of the population.

As we saw with Table 1, the results in Table S1 suggest that targeting with ML-based maps is most reliable in denser areas where more training data are available. However, it is important to note that the same holds true for other forms of geographic targeting. In particular, column 3 of Table S1 assesses the performance of the survey-based benchmark, based on imputed DHS estimates, in remote regions. Here, the dropoff in correlation with ground truth is much steeper. For instance, we observe that the correlation between imputed DHS estimates and NLSS ground truth in regions where the nearest DHS cluster is at least 10 kilometers away is just 0.233.

Counterfactual parity

Methodology. NLSS households are assigned to parity groups along each dimension based on their head of household. Each household is matched with replacement to another NLSS household whose head does not belong to the same demographic group. Matches are assigned using the nearest neighbor based on a propensity score that accounts for latitude, longitude, relative wealth index, and ward-level population density (a measure of urbanization) (2).

To calculate error terms, we imagine a situation in which every household in the country is targeted in order from poorest to wealthiest. Under perfect targeting, a household that is poorer than X% of other households will receive aid before X% of other households. Using this, a residual is calculated for each household j and targeting approach k:

$$\varepsilon_j^k = y_{wj} - y_{tj}^k \tag{1}$$

Here, y_{wj} is the percentile of household j's wealth index, and y_{tj}^k is the percentage of people targeted before household j using targeting approach k. Averaging these residuals over all households with heads belonging to a demographic group tells us whether members of this group are, on average, targeted earlier or later than they would be under optimal individual level targeting.

The same error metric is also calculated for each household's counterfactual. Comparing the mean error term between each demographic group and the corresponding counterfactual group establishes whether households in a demographic group are over- or under-targeted relative to similar households not in that demographic group. Comparing these differences between targeting approaches allows us to determine whether ML-based poverty maps exacerbate disparities relative to traditional survey-based approaches.

Results. For gender, religion, and age of head of household, results for all three data sources are quite similar (Figure S10). Variation is larger for language spoken, but with the exception of Igbo-speaking households ML-based targeting reduces the counterfactual disparity measure relative to survey-based targeting. Overall, results are consistent with the findings of our main parity specification that the ML-based poverty map does not in this case increase targeting disparities relative to the survey benchmark poverty map.

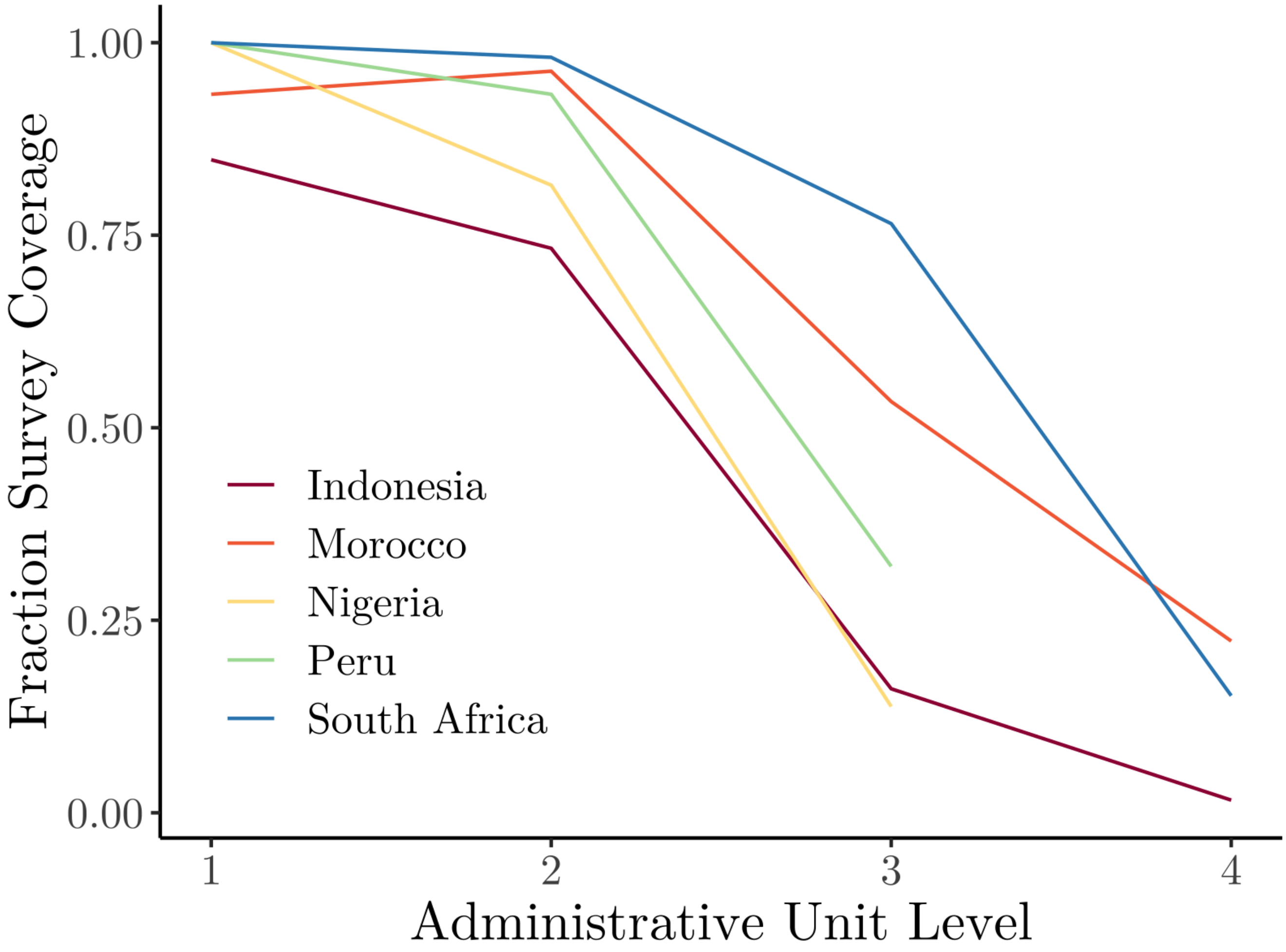

Fig. S1. Fraction of administrative units covered by Demographic and Health Survey data, for 5 LMICs

Notes: Based on nationally-representative Demographic and Health Survey (DHS) data from 5 LMICs, figure indicates the fraction of administrative units (from Admin-1 to Admin-4) for which the most recently conducted DHS contains at least one surveyed household. The number of administrative subdivisions varies by country.

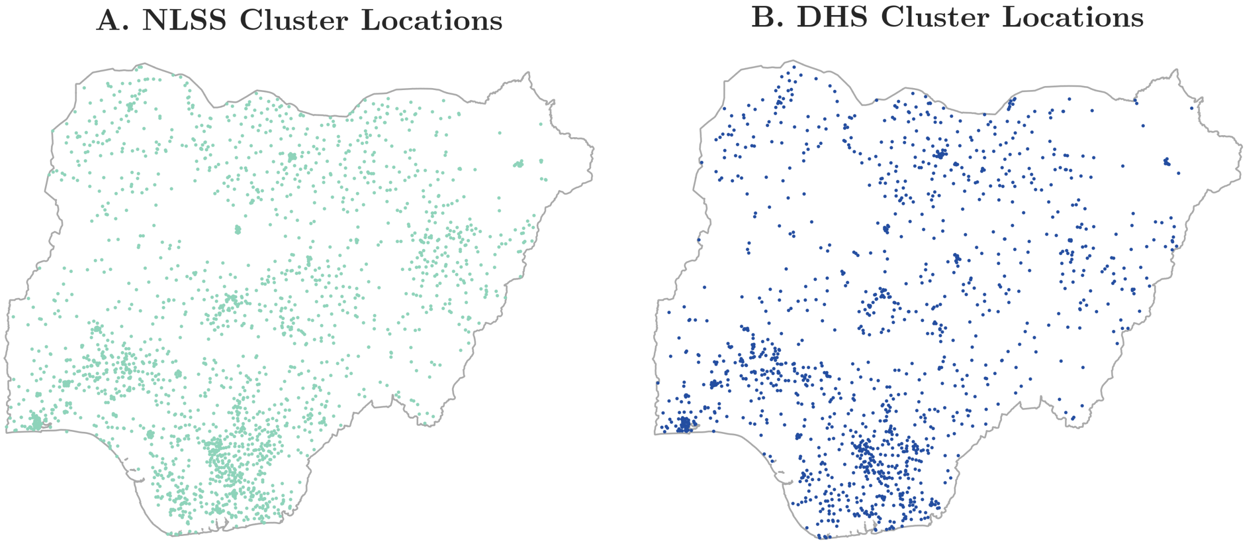

Fig. S2. Locations of clusters included in NLSS and DHS surveys

Notes: For privacy reasons, cluster locations are displaced by up to two kilometers in urban settings and up to five kilometers in rural ones. One percent of clusters, selected at random, are displaced by up to ten kilometers.

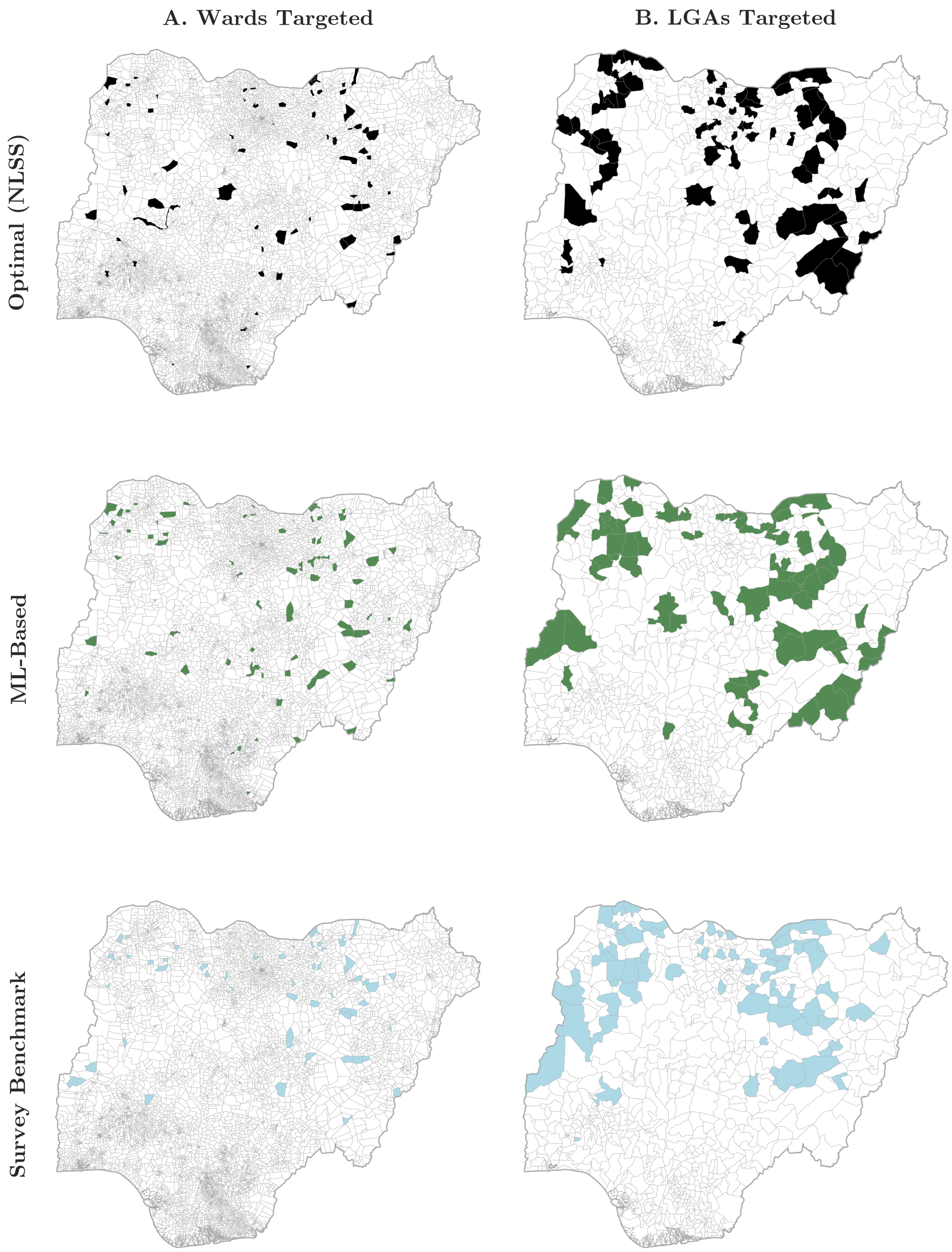

Fig. S3. Regions selected by each data source when 10% of the population is targeted

Notes: Three different datasets are used to rank wards (Panel A) and LGAs (Panel B) by poverty level. Units targeted when up to 10% of the population receives aid are highlighted. Population here is defined as the total population of the regions in our sample - that is, regions where both DHS and NLSS have data. This sample covers 77.1% of LGAs, home to roughly 148 million people, and 5.3% of wards, home to about 22.9 million people. Thus 10% of the population corresponds to 2.29 million people in Panel A, and 14.8 million people in Panel B.

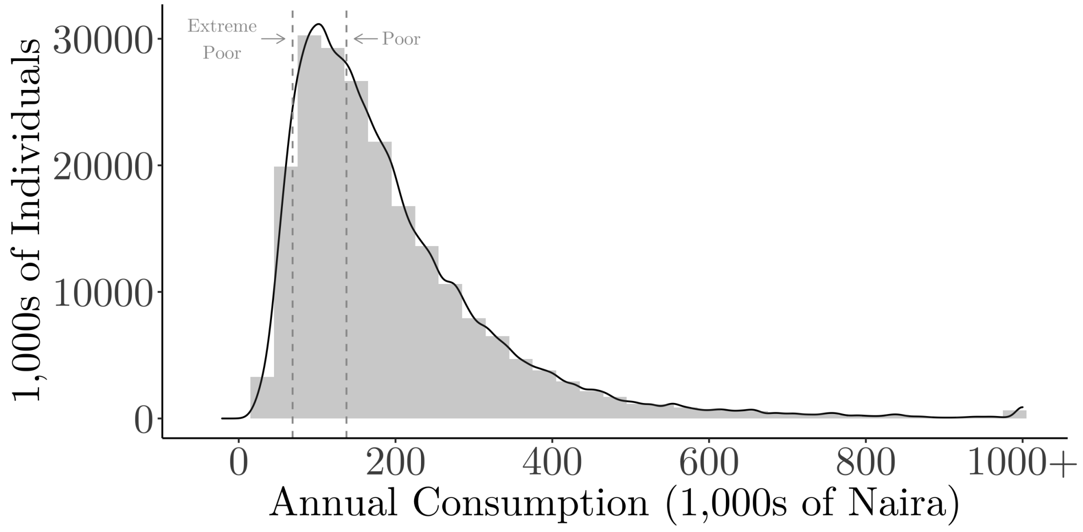

Fig. S4. Distribution of annual per capita consumption

Notes: Histogram of annual per capita consumption in thousands of Naira. Dashed lines show World Bank defined local thresholds for poverty (137,430 Naira, or roughly $1.05 USD per day in 2018) and extreme poverty (68,715 Naira, or roughly $0.53 USD per day in 2018).

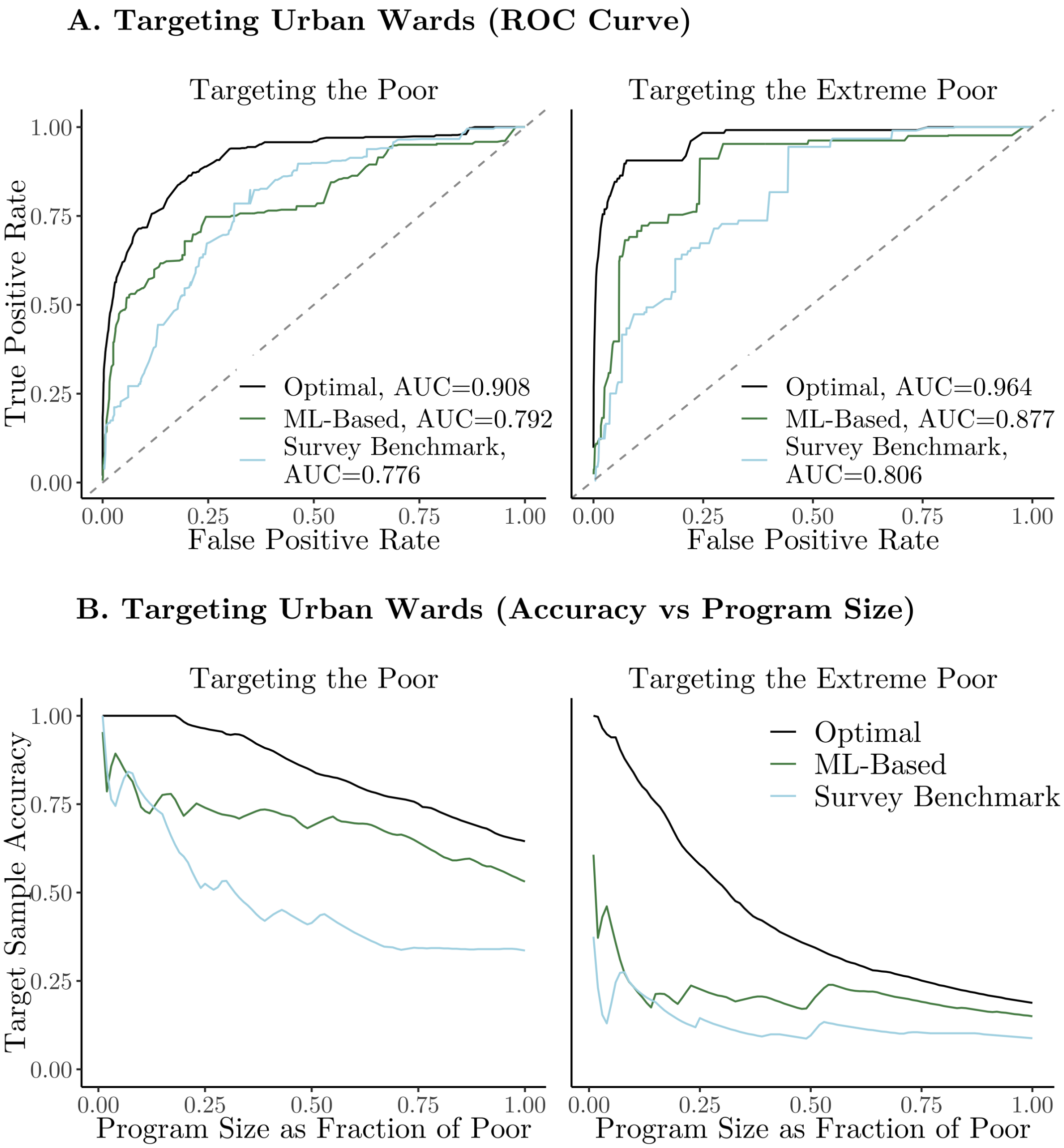

Fig. S5. Ward-level targeting performance, urban only

Notes: Three different datasets are used to identify the poorest urban wards; all residents of the selected wards are then targeted. Urban/rural ward classifications are obtained from Nigeria's National Bureau of Statistics. Panel A shows ROC curves based on whether the NLSS households in targeted wards are poor (left) or extreme poor (right). Panel B shows the fraction of program benefits going to the poor (left panel) and extreme poor (right panel) as the size of the anti-poverty program varies.

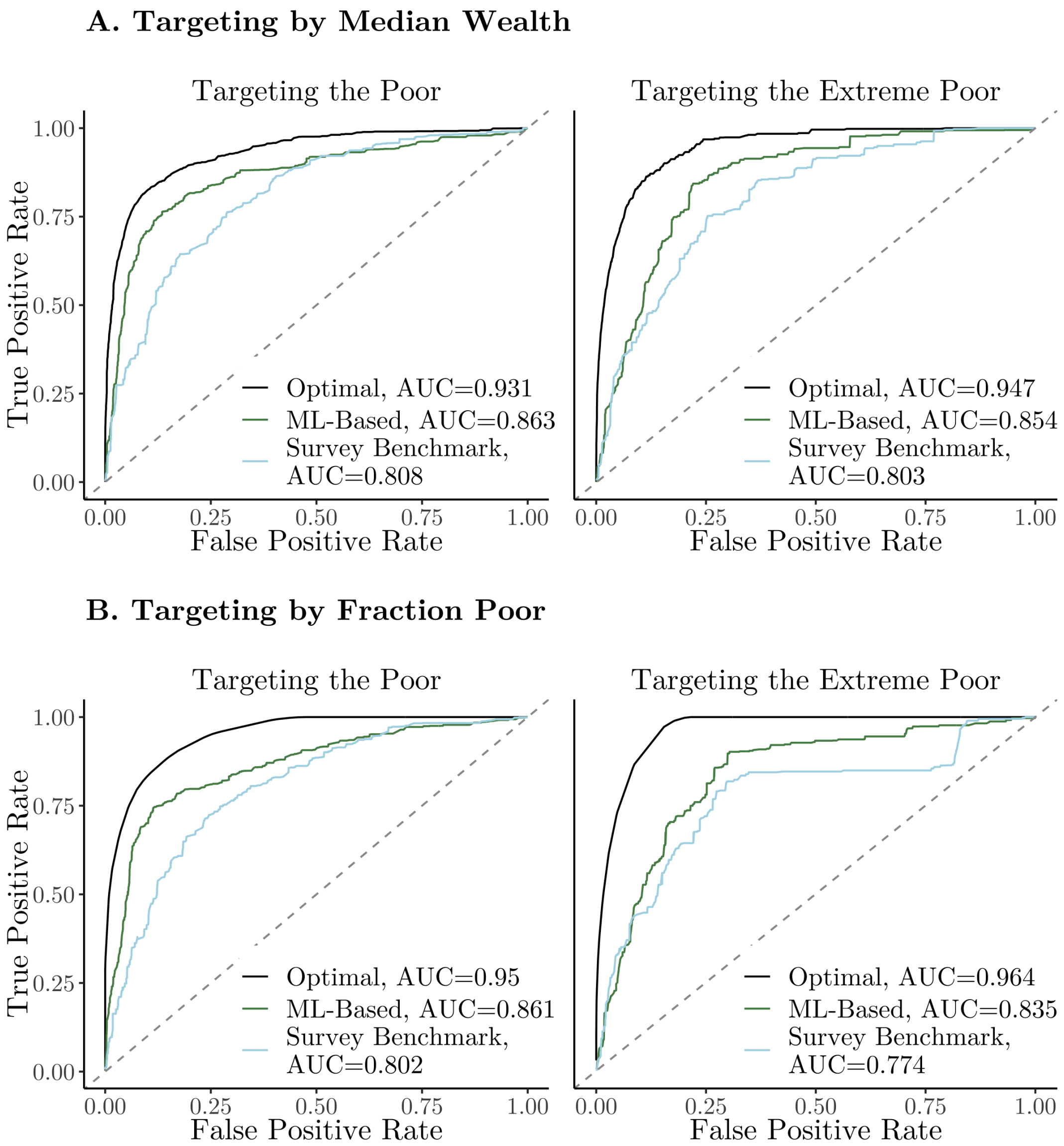

Fig. S6. Comparison of targeting performance for alternative targeting criteria

Notes: Three different datasets are used to rank wards by poverty level; resulting ROC curves are plotted. Panel A shows results when poverty level is estimated using median poverty. Panel B shows results when poverty level is estimated as the fraction of people in the region who are (extreme) poor. Targeting is evaluated based on whether NLSS households in targeted wards are poor (left) or extreme poor (right)

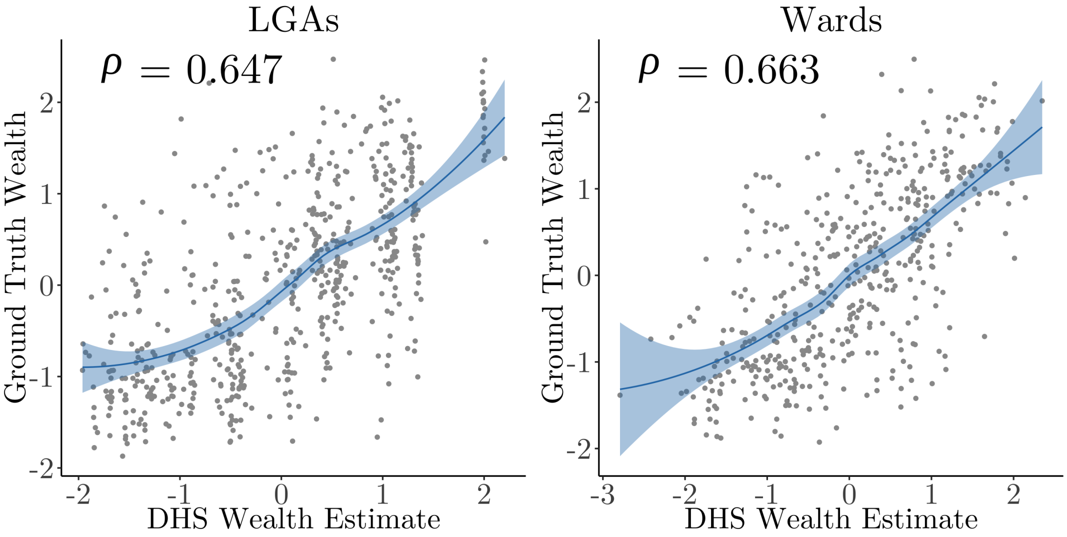

Fig. S7. Correlation between imputed DHS wealth indices and ground truth

Notes: Scatterplots compare the imputed survey benchmark (DHS) wealth estimates for each administrative unit against the ground truth (NLSS) wealth estimate. Wealth estimates are imputed using data from surrounding areas to simulate the accuracy of DHS in regions where it does not contain surveyed households; see section E.2 for details. Left plot shows Local Government Areas (LGAs), the Admin-2 unit, and the right plot shows Wards, the Admin-3 unit. All correlations are significant at p=0.001.

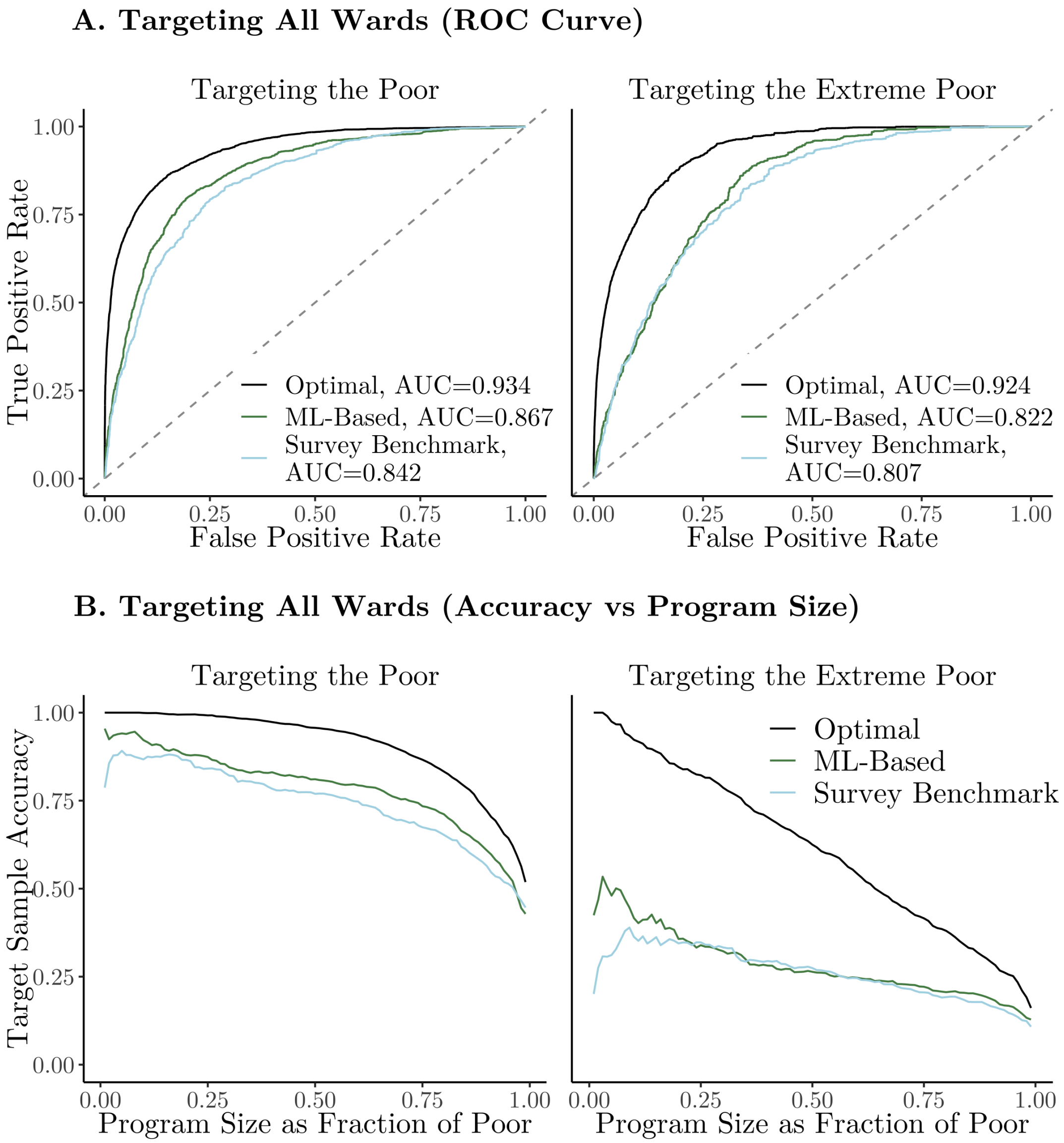

Fig. S8. Ward-level targeting performance when all NLSS wards are considered

Notes: Three different datasets are used to rank the poorest wards. All wards for which optimal (NLSS) data is available are considered. Wards where the benchmark survey (DHS) does not contain data are imputed using the process described in Section E.2. Panel A shows ROC curves based on whether the NLSS households in targeted wards are poor (left) or extreme poor (right). Panel B shows the fraction of program benefits going to the poor (left panel) and extreme poor (right panel) as the size of the anti-poverty program varies.

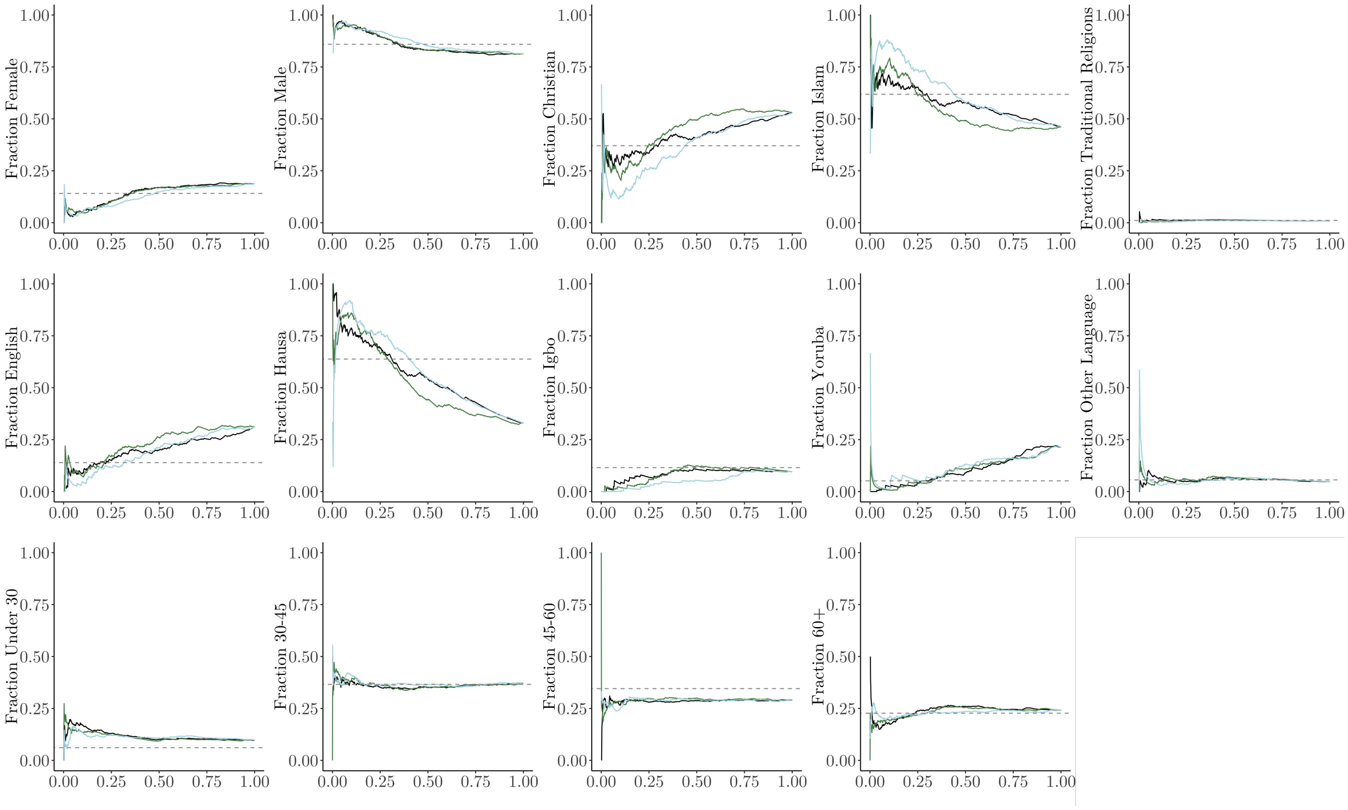

Fig. S9. Over- and under-targeting by demographic group as fraction of population targeted varies

Notes: Under perfect individual targeting, the fraction of transfers going to members of a demographic group would be equal to the fraction of total poor households belonging to that demographic group. Figure shows how the fraction of transfers going to each subgroup varies as a function of program size, as a fraction of total population. The dashed line shows the fraction of poor households in each demographic group – under perfect parity, this is the fraction of transfers that would go to members of this group.

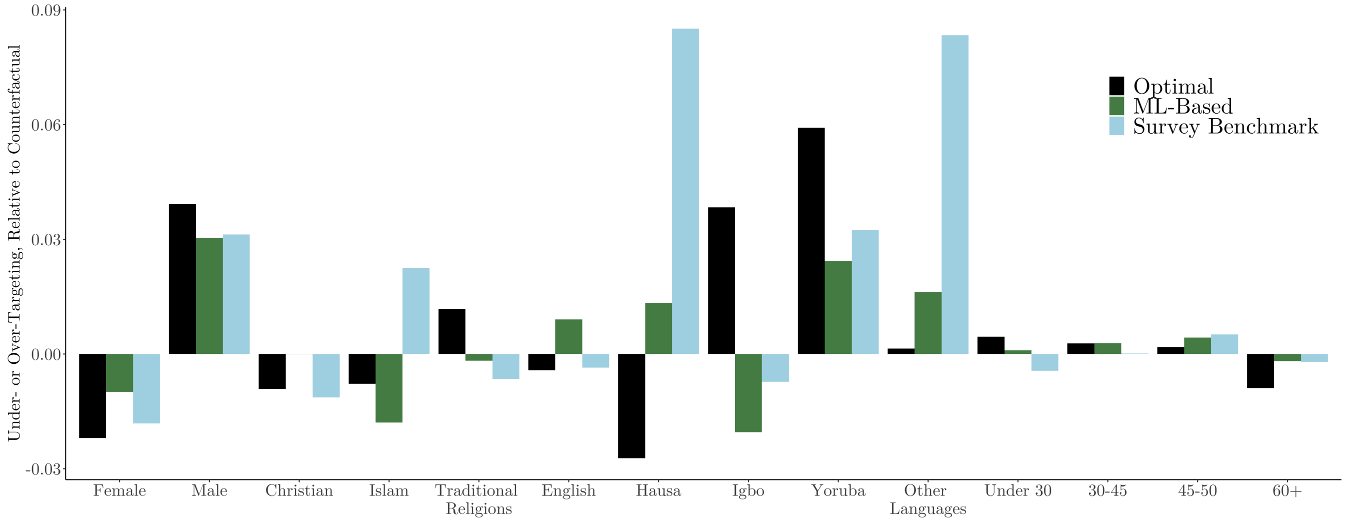

Fig. S10. Over- and under-targeting by demographic group relative to counterfactual

Notes: Households in each demographic group are matched with a similar household outside the demographic group using a propensity score. Figure shows the extent to which households in each demographic group are over- or under-targeted relative to the simulated counterfactual group. For each household, an error term is computed using their percentile in poverty rankings minus their percentile in targeting order; thus a negative error term implies the household receives aid too late in the targeting order, and a positive error term implies they receive aid too early. Plot shows the difference in mean error for demographic and counterfactual groups; that is, how much earlier or later on average households in this demographic group are targeted relative to similar households outside the demographic group.

	Number of Wards	Correlation with Ground Truth Estimates			
		ML-Based		*DHS-Based*	
1. All Wards with NLSS Data	2016	0.769	(0.751, 0.786)	0.676	(0.652, 0.699)
2. Wards without DHS Data	1552	0.759	(0.737, 0.779)	0.641	(0.611, 0.670)
Nearest DHS Cluster at Least:					
3. 1 km from Ward	1261	0.727	(0.700, 0.752)	0.580	(0.543, 0.616)
4. 5 km from Ward	643	0.629	(0.580, 0.674)	0.411	(0.345, 0.474)
5. 10 km from Ward	281	0.558	(0.472, 0.634)	0.233	(0.119, 0.341)
6. 15 km from Ward	118	0.613	(0.486, 0.715)	0.318	(0.145, 0.471)
7. 20 km from Ward	54	0.609	(0.408, 0.754)	0.200*	(-0.071, 0.444)

Table S1. Accuracy of ML-based poverty maps in regions with less DHS training data

Notes: The ML-based poverty map is trained using DHS data. This table reports the accuracy of the ML-based maps in regions where the nearest DHS cluster is progressively more distant. The first column indicates the number of wards that meet the criteria listed in the row heading. The second column indicates the correlation between ML-based and ground truth (NLSS) poverty estimates for this subset of wards, and the third shows this correlation using the DHS-based poverty map, with missing wards imputed. Row 1 considers all wards for which DHS and ground truth (NLSS) data are available. Row 2 considers wards with ground truth data but no DHS data. Rows 3-7 consider subsets of the wards in row 2 for which the nearest DHS cluster to the ward is at least some distance away. All correlations are significant at p=0.001 except where marked with * (DHS wards at least 20km from the nearest DHS cluster, p=0.146); parentheses show 95% confidence intervals.

References

1. NAO Nigeria, 1 million nigerians to benefit from covid-19 cash transfer programme. *Pulse Niger.* (2021).
2. L Kondmann, XX Zhu, Under the radar–auditing fairness in ml for humanitarian mapping. *arXiv preprint arXiv:2108.02137* (2021).
